# A MAPK-Driven Feedback Loop Suppresses Rac Activity to Promote RhoA-Driven Cancer Cell Invasion

**DOI:** 10.1371/journal.pcbi.1004909

**Published:** 2016-05-03

**Authors:** Joseph H. R. Hetmanski, Egor Zindy, Jean-Marc Schwartz, Patrick T. Caswell

**Affiliations:** Wellcome Trust Centre for Cell-Matrix Research, Faculty of Life Sciences, University of Manchester, Manchester, United Kingdom; University Hospital RWTH Aachen, GERMANY

## Abstract

Cell migration in 3D microenvironments is fundamental to development, homeostasis and the pathobiology of diseases such as cancer. Rab-coupling protein (RCP) dependent co-trafficking of α5β1 and EGFR1 promotes cancer cell invasion into fibronectin (FN) containing extracellular matrix (ECM), by potentiating EGFR1 signalling at the front of invasive cells. This promotes a switch in RhoGTPase signalling to inhibit Rac1 and activate a RhoA-ROCK-Formin homology domain-containing 3 (FHOD3) pathway and generate filopodial actin-spike protrusions which drive invasion. To further understand the signalling network that drives RCP-driven invasive migration, we generated a Boolean logical model based on existing network pathways/models, where each node can be interrogated by computational simulation. The model predicted an unanticipated feedback loop, whereby Raf/MEK/ERK signalling maintains suppression of Rac1 by inhibiting the Rac-activating Sos1-Eps8-Abi1 complex, allowing RhoA activity to predominate in invasive protrusions. MEK inhibition was sufficient to promote lamellipodia formation and oppose filopodial actin-spike formation, and led to activation of Rac and inactivation of RhoA at the leading edge of cells moving in 3D matrix. Furthermore, MEK inhibition abrogated RCP/α5β1/EGFR1-driven invasive migration. However, upon knockdown of Eps8 (to suppress the Sos1-Abi1-Eps8 complex), MEK inhibition had no effect on RhoGTPase activity and did not oppose invasive migration, suggesting that MEK-ERK signalling suppresses the Rac-activating Sos1-Abi1-Eps8 complex to maintain RhoA activity and promote filopodial actin-spike formation and invasive migration. Our study highlights the predictive potential of mathematical modelling approaches, and demonstrates that a simple intervention (MEK-inhibition) could be of therapeutic benefit in preventing invasive migration and metastasis.

## Author Summary

The majority of cancer-related fatalities are caused by the movement of cancer cells away from the primary site to form metastases, making understanding the signalling mechanisms which underpin cell migration and invasion through their local environment of paramount importance. Much has been discovered about key events leading to invasive cell migration. Here, we have taken this prior knowledge to build a powerful predictive model based on simple ON/OFF relations and logic to determine potential intervention targets to reduce harmful invasive migration. Interrogating our model, we have identified a negative feedback loop important to the signalling that determines invasive migration, the breaking of which reverts cells to a slower, less invasive phenotype. We have supported this feedback loop prediction using an array of in vitro experiments performed in cells within 2-D and physiologically relevant 3-D environments. Our findings demonstrate the predictive power of such modelling techniques, and could form the basis for clinical intervention to prevent metastasis in certain cancers.

## Introduction

An estimated 90% of cancer deaths are caused by metastatic secondary tumours [1], a process instigated as certain cells escape the primary tumour to migrate in, and invade through, the local micro-environment. Cancer cells can adopt a range of different migratory mechanisms to achieve such invasion [2]: some migrate in co-operation with near neighbours in whole sheet like structures or chains following initial ‘guerrilla’ cells [3], while others migrate individually, using distinct but interchangeable motility mechanisms. In most cases, the mechanisms which coordinate cell migration are dictated by Rho GTPases [4], of which Rac1 and RhoA are the most well-defined. Rho GTPases are molecular switches which can be in a GTP-bound 'on' state, or a GDP-bound 'off' state [5] in response to activating guanine nucleotide exchange factors (GEFs) and inhibiting GTPase activating-proteins (GAPs) [6]. Rac1 is considered the dominant GTPase acting at the leading edge of lamellipodia, polymerising actin via the Arp2/3 complex to form a dendritic actin network [7,8], while RhoA dominates at the rear of the cell to activate ROCK driven contractility and rear-retraction [8,9]. More recently, RhoA activity has been observed immediately at the leading edge in cells migrating in 2D, with Rac active in a zone immediately behind this [10]. Rac1 and RhoA are thought to be mutually antagonistic [11,12], and studies suggest that cyclic bursts of RhoA and Rac1 activity in a pseudo-oscillatory manner may drive the leading edge of some cells forward by producing a necessary push-pull mechanism [13,14].

In 3D and in vivo, single ‘mesenchymal’ cells, and leader cells in collective migration, migrate in a Rac-driven manner, and the mechanisms of actin polymerization, protrusion and force generation are thought to be analogous to lamellipodial migration in 2D [2,15,16]. However, lamellipodium-independent 3D migration strategies have also been identified. Single cells can adopt amoeboid modes of migration [2,17] and fibroblasts can move in an adhesion- and contractility-dependent lobopodial mode of migration [18,19]. Furthermore, Rab-coupling protein (RCP) dependent endocytic recycling of the fibronectin (FN) receptor α5β1 integrin promotes formation of filopodial actin-spike protrusions to drive invasive migration in FN rich 3D-ECM and in vivo in response to inhibition of αvβ3 integrin or expression of gain-of-function (GOF) mutant p53 in cancer cells [20,21].

Expression of gain-of-function mutant p53 (e.g. R273H, R175H), or inhibition of αvβ3 integrin (with small-molecule inhibitors, e.g. cRGDfV), promotes the association of RCP with α5β1 and leads to rapid recycling of this integrin to promote invasive migration [20,21,22]. Rather than directly influence the adhesive capacity of the cell, RCP and α5β1 recruit epidermal growth factor receptor 1 (EGFR1) to a recycling complex, controlling their co-trafficking to the tips of invasive pseudopods in motile cells [20]. This potentiates EGFR signalling specifically at the tips of invasive pseudopods, activating PKB/Akt which phosphorylates RacGAP1 to allow it’s recruitment to the leading edge by the cytoskleletal scaffold IQGAP1 [23]. RacGAP1 directly inactivates Rac1, allowing RhoA activation specifically at the front of invading cells [23], and RhoA-ROCK mediated activation of formin homology domain-containing 3 (FHOD3) leads to polymerisation of filopodial actin spike protrusions which promote motility in 3D ECM [24]. Whereas cells under basal conditions exhibit slow but directionally persistent migration in 2D and low invasiveness in FN-rich 3D-ECM, RCP-α5β1 trafficking promotes rapid, random migration in 3D and invasion in FN-rich ECM and in vivo [20,21]. Interestingly, mutant p53 expressing cancers are more metastatic in a number of contexts [21,25,26,27,28], and high RhoA activity has been observed at the leading edge of pancreatic cancer cells harbouring such mutations [29], suggesting that RhoA mediated protrusion could underlie the protrusive and invasive characteristics of a variety of metastatic tumours. Understanding the mechanisms by which the pro-invasive Rac1 to RhoA switch is potentiated at the leading edge of a polarised cell in further detail is of paramount importance to in turn identify potential therapeutic targets which may abrogate invasive cell migration leading to metastasis.

Given that RhoA and Rac1 are contained in a highly connected complex network that regulates their relative activities [30,31], it is likely that systems biology analyses will reveal regulatory interactions and feedback loops which are involved in altering GTPase activity and migratory phenotype. Boolean logic is an attractive modelling tool in the context of a large and essentially poorly characterised setting, since the detailed kinetic parameters (activation/inhibition rates, initial molecular concentrations) crucial to the generation of continuous models based on ordinary differential equations are not required [32,33]. Instead, the activity of all variables in the model is binarised into simple ON or OFF states, and all reactions are assumed to take the same arbitrary length of time. Boolean models can therefore encompass broad network topologies and order events to describe the underlying biology and predict outcomes and phenotypes in diverse cellular contexts, including cell migration [14,34]. Here we report a detailed model based on simple Boolean logic to connect EGF/EGFR1 to Rac1 and RhoA in the context of RCP/α5β1 trafficking, identifying nodes/directed links from stringent literature mining to build upon existing EGF signalling pathway models. This model accurately recreated existing experimental findings, and moreover identified a previously unanticipated negative feedback loop, involving MAP kinase dependent control of the Rac1 activating Sos1-Eps8-Abi1 complex which determines the RCP/α5β1 dependent Rac to RhoA switch. Hence, mathematical modelling has revealed a targetable aspect of RhoGTPase activity control, which could be exploited as an anti-invasive approach in mutant p53 expressing cancers.

## Results

### The hierarchy of Rac1 activators/inhibitors determines outcome in Boolean simulations

**Fig 1:**
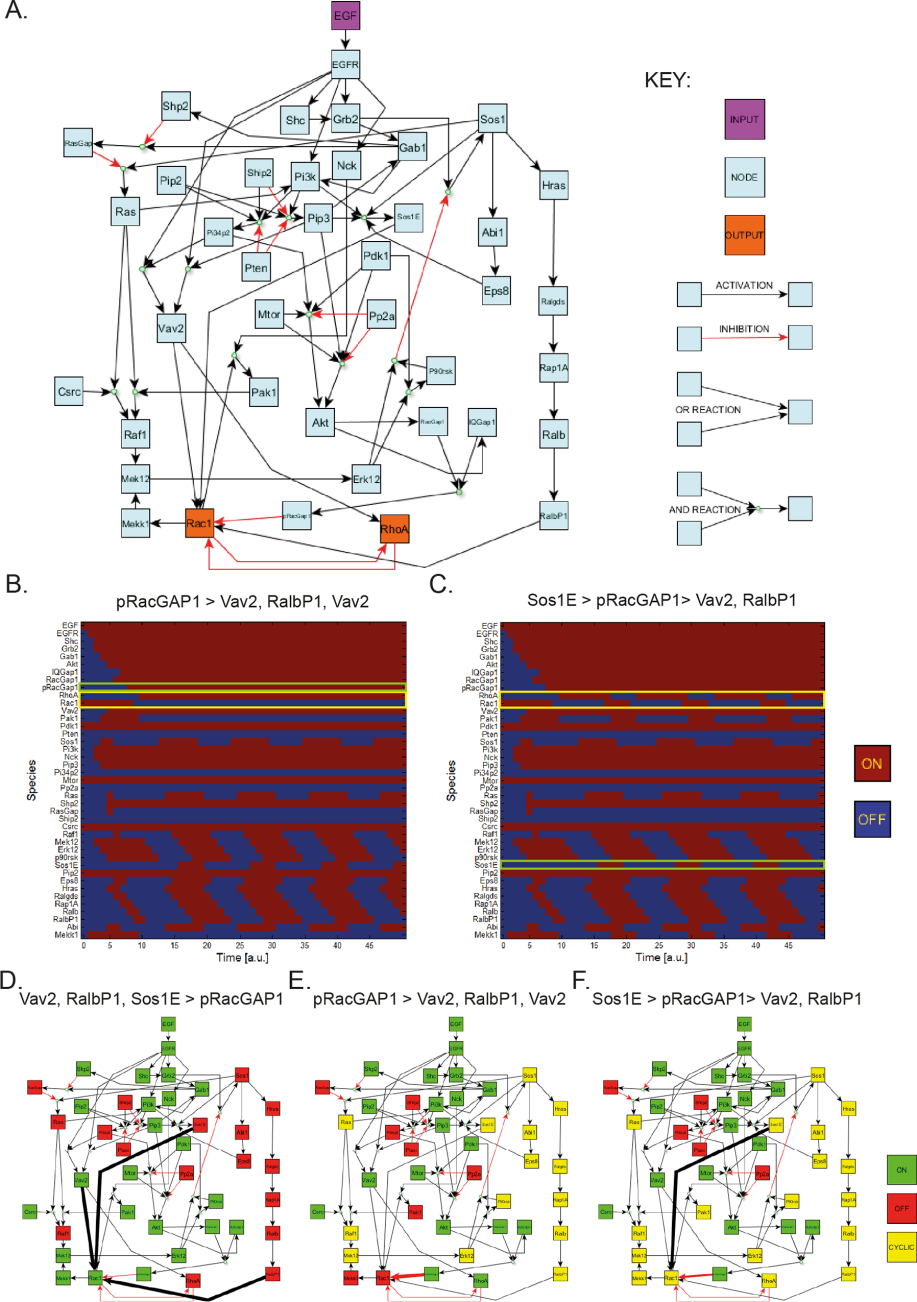
Logical simulation of integrin-driven cell migration. **A.** Reconstructed network describing signalling events leading to GTPase activity. The model consists of one input node, EGF, two nominated output nodes, Rac1 and RhoA, and 38 intermediate nodes. Reactions included in the model are activation or inhibition, where some reactions need cooperation of two or more upstream nodes via AND gates. See S1 Table for references to all reactions included in the model. **B, C.** Time-course simulation outputs for the first 50 time increments of the model for different Rac1 activator/inhibitor hierarchies, where the outputs of interest Rac1 and RhoA (yellow box) and the dominant Rac1 activator/inhibitor (green box) are highlighted: **B.** pRacGAP1 dominates Rac1 activity above all Rac1 activator. As pRacGAP1 activity is ‘switched’ ON, Rac1 activity is ‘switched’ OFF after one time increment, RhoA activity is ‘switched’ ON a further time increment later and Rac1/RhoA remain OFF/ON respectively as t → ∞; **C.** Sos1E dominates Rac1 activity over pRacGAP1 which in turn dominates Rac1 activity over Vav2 and RalbP1. Initially pRacGAP1 activation switches OFF Rac1 which switches on RhoA later as before, however when Sos1E is ‘switched’ ON (green box), Rac1 is ‘switched’ ON after one time increment and then RhoA is ‘switched’ OFF after one further time increment. When Sos1E is later ‘switched’ OFF, Rac1 and subsequently RhoA are switched OFF/ON respectively, leading to cyclic activity of Rac1 nd RhoA as t → ∞. **D-F.** Steady-state outputs of the model for simulations with example different Rac1 activator/inhibitor hierarchies (full list of hierarchies in S3 Table), where activator/inhibitor dominance is visualised by reaction arrow thickness: **D.** All Rac1 activators Vav2, RalbP1 and Sos1E (Sos1-Eps8-Abi1 complex) (thick black arrows) dominate Rac1 activity over the Rac1 inhibitor pRacGAP1 (thin red arrow); **E.** pRacGAP1 (thick red arrow) dominates Rac1 activity over Vav2, RalbP1 and Sos1E (thin black arrows); **F.** Sos1E (thick black arrow) dominates Rac1 activity over pRacGAP1 (medium red arrow) which in turn dominates Rac1 activity over Vav2 and RalbP1 (thin black arrows). Note steady-state outputs in the Boolean simulations can only be stable activity where the node is ON for all time as t → ∞ (green), stable inactivity where the node is OFF for all time as t → ∞ (red) and cyclic activity where the node is encapsulated in a stable limit cycle and cycles regularly between ON and OFF activity as t → ∞ (yellow). All simulations performed in CellNetAnalyzer.

RCP and α5β1 recruit EGFR1 into a recycling complex, and promotes the return of this receptor to the plasma membrane where it can re-engage ligand to amplify the EGFR1 signalling. Because the signalling response (PKB/Akt and RhoA activation, Rac inactivation) localises to the front of the cell [23], we considered signalling only within this domain, and modelled the EGFR signalling network from the initial stimulation with EGF (the external input) to the Rac and Rho GTPases (the nominated outputs) by generating a Boolean network that included over 40 nodes and interactions mined from the literature (curated as described in Methods; Fig 1A). At the initiation of signalling within the model, PDK1, mTor, c-Src, Pip2 and Rac1 are ON, as this is the assumed activity in basal conditions prior to EGF signalling. All other nodes were initially set to OFF. Under these conditions the first 50 time increments were simulated to assess the effect on the RhoA and Rac1 outputs to allow for steady state or steady limit cycle to be reached (Fig 1B and 1C).

**Fig 2:**
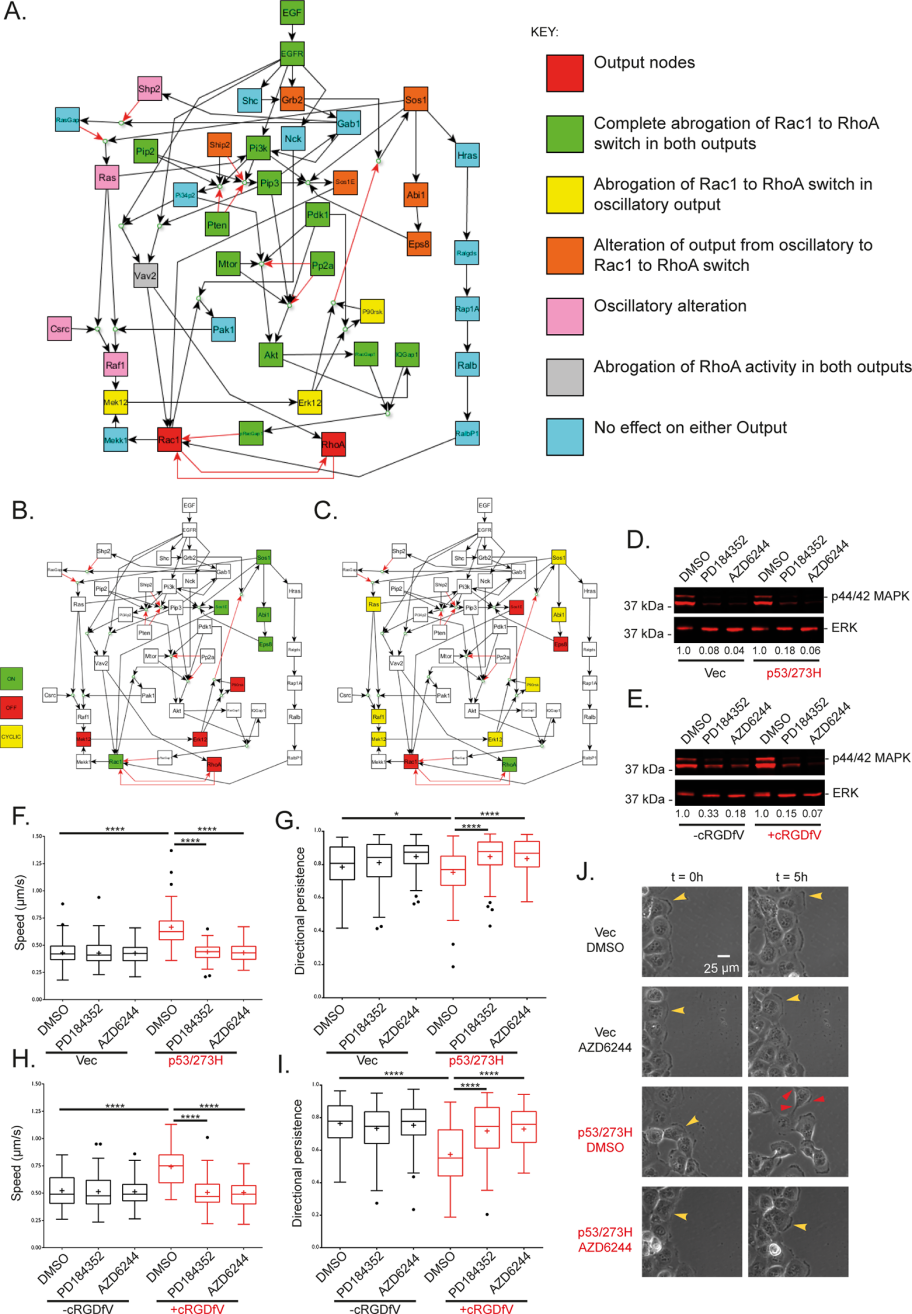
*In silico* node knockouts predict a Sos1/Ras/Raf/MEK1/2/ERK1/2/p90RSK negative feedback loop affects Rac1 and RhoA dynamics via the Rac1 activation Sos1-Eps8-Abi1 complex. **A.** Effect of individual *in silico* node knockout of every non-output node in the model on RhoA/Rac1 output dynamics. Node knockouts were performed by removing all inward edges for each node individually such that the knocked out node remained OFF for all time, except for inhibitory nodes with no input edges PTEN, Ship2 and PP2a which were set to ON. Note ‘Rac1 to RhoA switch’ output corresponds to Fig 1B and 1E and cyclic output corresponds to Fig 1C and 1F. **B-C.** Selected steady-state node activity for the Sos1E > pRacGAP1 > RalbP1, Vav2 cyclic activity inducing hierarchy for proteins affecting Rac1 and RhoA dynamics given knockout of **B** MEK1/2 and **C** Eps8. MEK1/2 is a critical node in the Sos1/Ras/Raf/MEK1/2/ERK1/2/p90RSK negative feedback loop, whereby removal of MEK1/2 keeps ERK1/2 and p90RSK inactive, which removes the inhibition of Sos1 keeping the Sos1-Eps8-Abi1 complex active which keeps Rac1 active and RhoA inactive and abrogates the pro-invasive Rac1 to RhoA switch. Eps8 removal prevents formation of the Sos1-Eps8-Abi1complex which prevents activation of Rac1 regardless of activity state of Sos1/Ras/Raf/MEK1/2/ERK1/2/p90RSK negative feedback loop nodes. **D-E.** Western blots showing steady state endogenous levels of phosphorylated p44 and p42 MAP Kinase (Erk1 and Erk2) and total levels of ERK2 (for loading) for cells treated with a single MEK1/2 inhibitor, PD184352 or AZD6244, or DMSO for vehicle for different cell types: **D.** H1299s, a non-small lung cell carcinoma, either stably expressing mutant p53 (right) or with an empty for p53 (left); **E.** A2780s, an epithelial ovarian cancer cell line, with (right) and without (left) stimulation with cRGDfV. Numbers below each band denote the intensity level normalised to the corresponding DMSO control. **F-J.** Effects of MEK1/2 inhibition on 2-D cell migration in >10 hour scratch wound experiments. **F.** Average speed of tracked H1299 cells over the time taken for wounds to close or 16 hours (whichever comes first). Cells expressing mutant p53 or with an empty vector for p53 were treated with DMSO or MEK1/2 inhibitors ~1 hour prior to imaging. **G.** Average persistence of the same tracked cells as in F. persistence is the measure of the distance between the cells first time-point position and last time-point position divided by the total distance travelled by cell between every time point position. **H.** Average speed of tracked A2780s cells for the same conditions as in F, cells were treated with cRGDfV at the same time as DMSO/MEK1/2 inhibitors ~1 hour prior to imaging. **I.** Average persistence for same tracked A2780 cells. Data are representative of 3 experiments, and 90 cells were tracked per condition. Graphs are Tukey boxplots where + represents the mean of each condition. **** indicates one way ANOVA with post-hoc Tukey HSD with p-value < 0.0001, * indicates p-value < 0.05 **J.** Representative images of H1299 cells in a sub-domain of an image at t = 0 (the initial frame) and the same sub-domain at t = 5 hours (30 10 minute frames later), for cells without/with mutant p53 expression and treated with DMSO or MEK1/2 inhibitor AZD6244. Yellow arrow heads indicate lamellipodial leading edge actin, while red arrow heads indicate more spike-like protrusions. The same cell is highlighted with arrow heads at t = 0 and t = 5 hours for each different condition.

Simulation results depended *a priori* on the unknown hierarchical binding affinity of the Rac1 activators/inhibitors. In general, a GEF and a GAP can never bind to and activate/inactivate a Rho GTPase in an identical spatiotemporal state [6]. For example, if the Rac inhibitor preferentially binds to Rac1 compared to the Rac activator, then the Rac inhibitor must be OFF for the Rac activator to activate Rac1. Three Rac1 activators were included in the model: Vav2, RalBP1 and Sos1E (a moniker used to describe the Sos1-Eps8-Abi1 complex which shows Rac-specific GEF activity [35]); and one Rac inhibitor: the phosphorylated form of RacGap1 (which is localised to the leading edge upon RCP-driven integrin/EGFR trafficking and hence positioned to inactivate Rac [23]). All logical hierarchies involving the Rac1 activators and inhibitor and subsequent outputs are summarised in S3 Table. Hierarchies exhibited three distinct categories of RhoA/Rac1 binary output dynamics (Fig 1D–1F): 1) Rac1 remains ON and RhoA remains OFF for all time increments (Fig 1D); 2) cyclic bursts of RhoA and Rac1 activity (Fig 1F); and 3). Rac1 switches OFF, RhoA switches ON and these activities are sustained, Fig 1E). If Vav2, in any combination with the other activators, affected Rac1 more readily than RacGAP1 then Rac1 remained active for all time, and due to the mutual antagonism between the two different GTPases, RhoA remained inactive (Fig 1D), which contradicts the premise that RCP-mediated α5β1-EGFR1 co-trafficking promotes a pro-invasive Rac1 to RhoA switch in conjunction with EGF stimulation. This hierarchy was therefore rejected based on modelling results. Logical relations RalBP1 > pRacGAP1 > Vav2, Sos1E, RalBP1 > pRacGAP1 > Vav2 and Sos1E > pRacGAP1 > Vav2, RalBP1 all gave rise to outputs exhibiting both Rac1 and RhoA activity in a cyclic manner. Crucially however only the relation Sos1E > pRacGAP1 > Vav2, RalBP1 exhibited clear RhoA dominance in the output, whereby RhoA activity is ON for significantly more time increments than Rac1 (1C, F). RalBP1 > pRacGAP1 > Vav2, Sos1E and Sos1E, RalBP1 > pRacGAP1 > Vav2 did not exhibit sufficient RhoA activity to agree with previous findings [23] so was rejected as a plausible output. Finally, the hierarchy in which RacGAP preferentially inactivates Rac1 induced a simple Rac1 to RhoA switch (Fig 1B and 1E) which was a further permissible output to describe the known GTPase dynamics.

### *In silico* node knockouts and prediction classifications

The two different plausible sets of output dynamics are in close agreement with previous experimental findings. Whether there is simply a switch output in Rac1 to RhoA activity at the leading edge during RCP-driven invasive cell migration (because Rac inactivation dominates over Rac activators) or whether the migration is driven by RhoA dominant pseudo-oscillatory Rho/Rac dynamics at the leading edge (because Sos1-Eps8-Abi1 complex preferentially affects Rac1) as has been suggested for some migratory schemes [14] is not known. We therefore performed individual *in silico* node knockouts, wherein each node was set to an OFF value for all time points by removing all edges leading into said node (or turning the nodes ON in the case of the inhibitory proteins SHIP2, PTEN and PP2a), to ascertain the effect on the RhoA and Rac1 dynamics for both the Sos1E > pRacGAP1 > Vav2, RalBP1 hierarchy (the cyclic pseudo-oscillatory output) and the pRacGAP1 > Sos1E, RalBP1, Vav2 hierarchy (the switch output) (Fig 2A).

For both hierarchies, the *in silico* knockouts accurately mimic previous experimental evidence: knockout of the node IQGAP1 for example results in persistent Rac1 ON and RhoA OFF activities, as seen previously in FRET-FLIM experiments in IQGAP1 knockdown cells [23]. Furthermore, upstream activators of PKB/Akt are an absolute requirement for plausible Rac1/RhoA outcomes (Fig 2A). Further to replicating known priors regarding certain proteins contained within the network, the node knockout workflow made predictions for the perturbation effect of all nodes in the model. For the pRacGAP1 > Sos1E, RalBP1, Vav2 case, the only proteins which are implicated as vital for the pro-invasive Rac to Rho switch downstream of RCP-dependent trafficking and subsequent EGF stimulation were those which act upstream of Akt activation, such as the well-known Akt activators PI3K and PIP3 [36,37] or the proteins upstream of RhoA activation, which in this model were Vav2 and its activator PIP3 (Fig 2A). For the pseudo-oscillatory inducing Sos1E > pRacGAP1 > Vav2, RalBP1 hierarchy the *in silico* knockouts provided more diverse predictions. As well as the same predictions concerning proteins upstream of Akt and RhoA, a Sos1 negative feedback loop was also predicted to affect GTPase dynamics (Fig 2A). Sos1 initiates a signalling path in which Ras [38], Raf1 [39], MEK1/2 [40], ERK1/2 [41] and p90RSK (in the presence of active PDK1 [42]) become active. Serine/threonine phosphorylation of Sos1 by active ERK1/2 or p90RSK however has been shown to cause dissociation of Sos1 from Grb2-EGFR and down-regulate Sos1 activity [43], which in Boolean terms translates to active ERK1/2 or p90RSK inhibiting Sos1 (i.e. switching it from ON to OFF). Once Sos1 is switched OFF, all proteins activated downstream of Sos1 are switched OFF, including the Sos1-Eps8-Abi1 complex (therefore preventing activation of Rac1) as well as all the proteins involved in the Ras, Raf1, MEK1/2, ERK1/2, p90RSK pathway. Once both ERK1/2 and p90RSK are OFF, Sos1 activation is allowed and the negative feedback loop initiates again. Breaking the feedback loop at different points *in silico* elicited altered GTPase activity: if MEK1/2 was removed, Sos1 remained ON, hence the Sos1-Eps8-Abi1 complex remained ON to keep Rac1 ON, hence there was no Rac1 to RhoA switch (Fig 2B); however if Eps8 was removed, the GTPase output changed from cyclic pseudo-oscillations to a Rac1 to RhoA switch as the Sos1-Eps8-Abi1 complex no longer formed, and thus the MEK1/2-driven feedback loop no longer had any effect on Rac1 activation (Fig 2C). This provided testable predictions which could be used to determine both the veracity of the model described and the hierarchy of GEF and GAP activity within the Boolean network.

To test model robustness and determine that the RhoA dominant cyclic activity of Rac1 and RhoA was not caused by the synchronous updating scheme used in Boolean simulations, we introduced inherent stochasticity into the model by using random asynchronous updates (whereby in any time increment, the next executable reaction will be chosen at random). Results using this updating scheme were in close agreement with the oscillations observed previously (Fig 1C), whereby the crucial RhoA dominance that has been observed experimentally remained present in all asynchronous simulations (S1A Fig) and all simulations showed some cyclic RhoA and Rac1 activity (S1B Fig). Moreover, all predictions concerning MEK1/2 and Eps8 perturbation remained intact. This verification shows that the synchronous update algorithm used in our Boolean simulations does not affect the core findings of the simulations or cause spurious oscillations.

### MEK1/2 inhibition promotes phenotypic reversion to slow lamellipodial migration in 2-D and 3-D environments

Modelling results predicted that if the Sos1-Eps8-Abi1 complex preferentially affects Rac1, then removal/inhibition of MEK1/2, ERK1/2 or p90RSK will abrogate the invasive migration driven by the Rac1 to RhoA switch. To test this prediction experimentally we imaged cells migrating into a 2-D scratch-wound in the presence or absence of MEK1/2 inhibitors in two different carcinoma cell lines: A2780s, an ovarian cancer cell line where RCP driven α5β1/EGFR trafficking was promoted by inhibition of αvβ3 with cRGDfV; and H1299s, a non-small cell lung cancer cell line in which α5β1/EGFR trafficking was promoted via mutant p53 expression. For both cell lines, both PD184352 and AZD6244, two mechanistically different MEK1/2 specific inhibitors [44,45], showed clear suppression of p44/42 MAPK phosphorylation (Fig 2D and 2E). Under basal conditions, cells migrated slowly and persistently (Fig 2F–2I) with broad lamellipodia at the leading edge (Figs 2J and S2A, S1 Movie) consistent with high levels of active Rac at the leading edge. However when RCP-driven α5β1 trafficking was promoted, cells exhibited a more rapid and random migratory phenotype (Fig 2F–2I, S1 Movie) with narrow ruffling leading edge protrusions (Figs 2J and S2A, S1 Movie) rather than Rac-driven lamellipodia, consistent with suppression of Rac activity. MEK1/2 inhibition had no effect on speed, persistence or protrusion morphology under basal conditions (Figs 2F–2J and S2A, S1 Movie). However, in the context of RCP-mediated integrin-RTK co-trafficking and signalling, MEK1/2 inhibition reduced speed and increased persistence levels comparable to those seen under basal conditions in both A2780 and H1299 cell models (Fig 2F–2I). The phenotypic reversion was also evident in protrusion morphology, whereby narrow ruffling protrusions were replaced with broad lamellipodial structures (Figs 2J and S2A, S1 Movie), consistent with activation of Rac as predicted by the Boolean model with the Sos1E > pRacGAP1 > Vav2, RalBP1 hierarchy. Moreover, this suggests that a simple Rac to RhoA switch does not accurately describe the dynamics of RhoGTPase activity in invading cancer cells.

**Fig 3:**
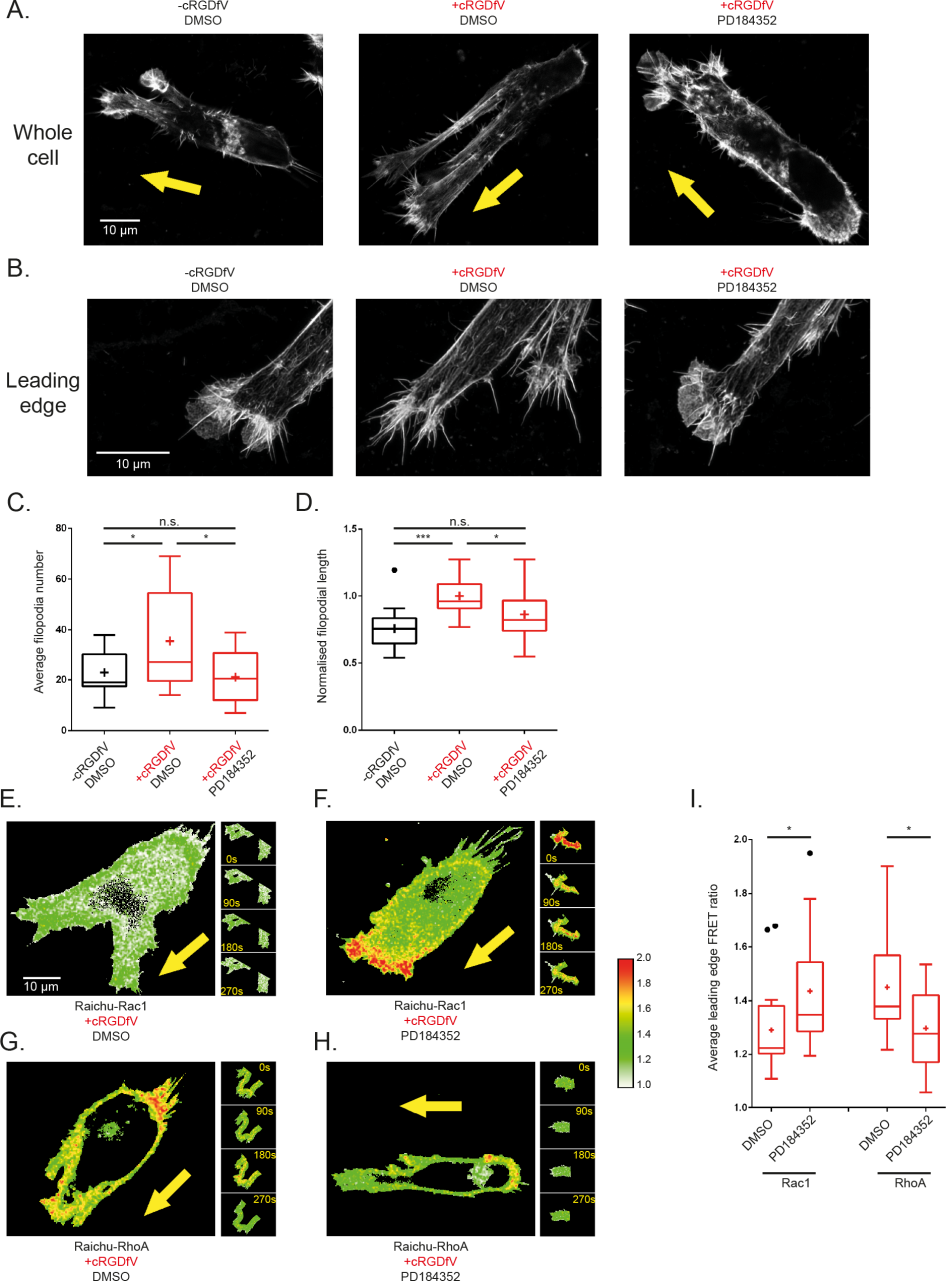
MEK1/2 inhibition promotes invasive-opposing leading edge lamellipodial structures in 3-D by increasing localised Rac1 activity and decreasing localised RhoA activity. **A** Whole A2780 cells seeded on cell derived matrix and stained with Alexa Fluor 488 Phalloidin, imaged by confocal microscopy to reveal actin structures of cells arrested in position during migration subject to different treatment conditions: (left) a cell without cRGDfV stimulation and treated with DMSO ~2 hours prior to fixing exhibiting a more 3-D lamellipodial actin structure; (centre) a cell treated with cRGDfV and DMSO for vehicle ~2 hours prior to fixing exhibiting more pseudopodia and filopodia at the leading edge; (right) a cell treated with cRGDfV and PD184352 ~2 hours prior to imaging exhibiting a reversion back to a more lamellipodial leading edge. **B.** High resolution maximum Z projection images of the leading edge actin structures of cells fixed and stained as in A. A 0.7 Airy pinhole was used to delineate individual filopodia, with a zoom factor of 4.0 on a 100x objective for cells treated: (left) without cRGDfV, with DMSO which exhibits fewer, shorter filopodial protrusions and abundant actin veiling; (centre) with cRGDfV and DMSO for vehicle, which exhibits more, longer filopodia and little actin veiling; (right) with cRGDfV and PD184352 which exhibits a reversion to the basal phenotype with fewer, shorter filopodia with increased actin veiling. **C-D.** Average **C.** number and **D**. normalised length of filopodia per leading edge of fixed, polarised cells in maximum projection high resolution confocal Z-stack images under treatment conditions as indicated. N > 13 cells per condition across 3 experimental repeats, one way ANOVA with Holm-Sidak post hoc test, * indicates p<0.05, *** p<0.001. **E-H** Representative Ratiometric FRET images of whole A2780 cells on CDMs at single timepoints (main images) and leading edge quantified areas (panel of images, right). **E.** Cell transfected with Raichu-Rac1 probe, stimulated with cRGDfV and treated with DMSO for vehicle; **F.** Cell transfected with Raichu-Rac1 probe, stimulated with cRGDfV and treated with PD184352; **G.** Cell transfected with Raichu-RhoA probe, stimulated with cRGDfV and treated with DMSO for vehicle; **H.** Cell transfected with Raichu-RhoA probe, stimulated with cRGDfV and treated with PD184352. All images have the same custom look-up table (LUT) applied and set between 1.0 and 2.0 (shown, right of images), where red pixels denote high GTPase activity. **I.** Quantification of average FRET ratio in the leading edge of all analysed cells across all 20 timepoints in each 5 minute movie. N > 15 cells across 3 experimental repeats. Tukey boxplot used with mean indicated as +. Student t-tests used, * indicates p <0.05.

Imaging of fixed A2780 cells within a 3D cell-derived matrix (CDM) environment at high magnification enabled the resolution of F-actin structure in migrating cells. In line with previous findings [24], and analogous to 2-D scratch wound data, cells switch from lamellipodial protrusions (Fig 3A and 3B) upon induction of RCP/α5β1 trafficking with cRGDfV to actin-spike protrusions which are made up of numerous long filopodia which lack veils of dendritic actin (Fig 3A and 3B). Strikingly, upon inhibition of MEK1/2, protrusions revert back to lamellipodia with fewer and shorter filopodia similar to the leading edge structures seen in the basal, unstimulated case (Fig 3A, 3B, 3C and 3D). These data demonstrate that MEK inhibition prevents the establishment of actin-spike protrusions that form as a consequence of RCP-mediated trafficking of α5β1 and EGFR1, and suggest that in this context cells are unable to implement the Rac to RhoA switch in a physiologically relevant 3-D environment.

### MEK1/2 inhibition promotes Rac1 activation and supresses RhoA activity at the leading edge of motile cells in 3-D micro-environments

To investigate whether the observed MEK1/2 inhibition phenotypes, slowing migration and promoting stable lamellipodial protrusions, were indeed via an effect on the Rho GTPases Rac1 and RhoA as predicted by the model, we used FRET based biosensor experiments for cells individually migrating in 3D CDM. We developed a method to determine the mean average FRET ratio readout for the leading edge of motile cells by isolating a ‘ring’ 40 pixels wide in the front 25% of each cell (S6 Fig, also see Methods). Using this unbiased quantification method, we could show that Rac activity was low at the leading edge of cells moving on 3D ECM, whereas RhoA activity was high when RCP-driven trafficking of α5β1 and EGFR1 was promoted in A2780 cells upon cRGDfV treatment (Fig 3E, 3G and 3I, S2 Movie) or in H1299 cells upon expression of mutant p53 (S3A, S3C and S3E Fig), in agreement with our previous observations [23]. However, upon MEK1/2 inhibition with PD184352, Rac1 activity at the leading edge of a cell was significantly increased (Figs 3F and 3I and S3B and S3E, S2 Movie) whereas RhoA activity at the cell front (but not rear) was reduced (Figs 3H and 3I and S3D and S3E, S2 Movie). These data directly demonstrate that, in agreement with the model, inhibition of MEK prevents the Rac to RhoA switch observed in cells migrating in an RCP/ α5β1/EGFR1 driven manner, and suggest that MEK-ERK signalling could indeed provide a feedback loop to periodically supress Rac activity and permit RhoA activation.

### Eps8 is required for sensitivity to MEK1/2 inhibition in 2-D cell migration

**Fig 4:**
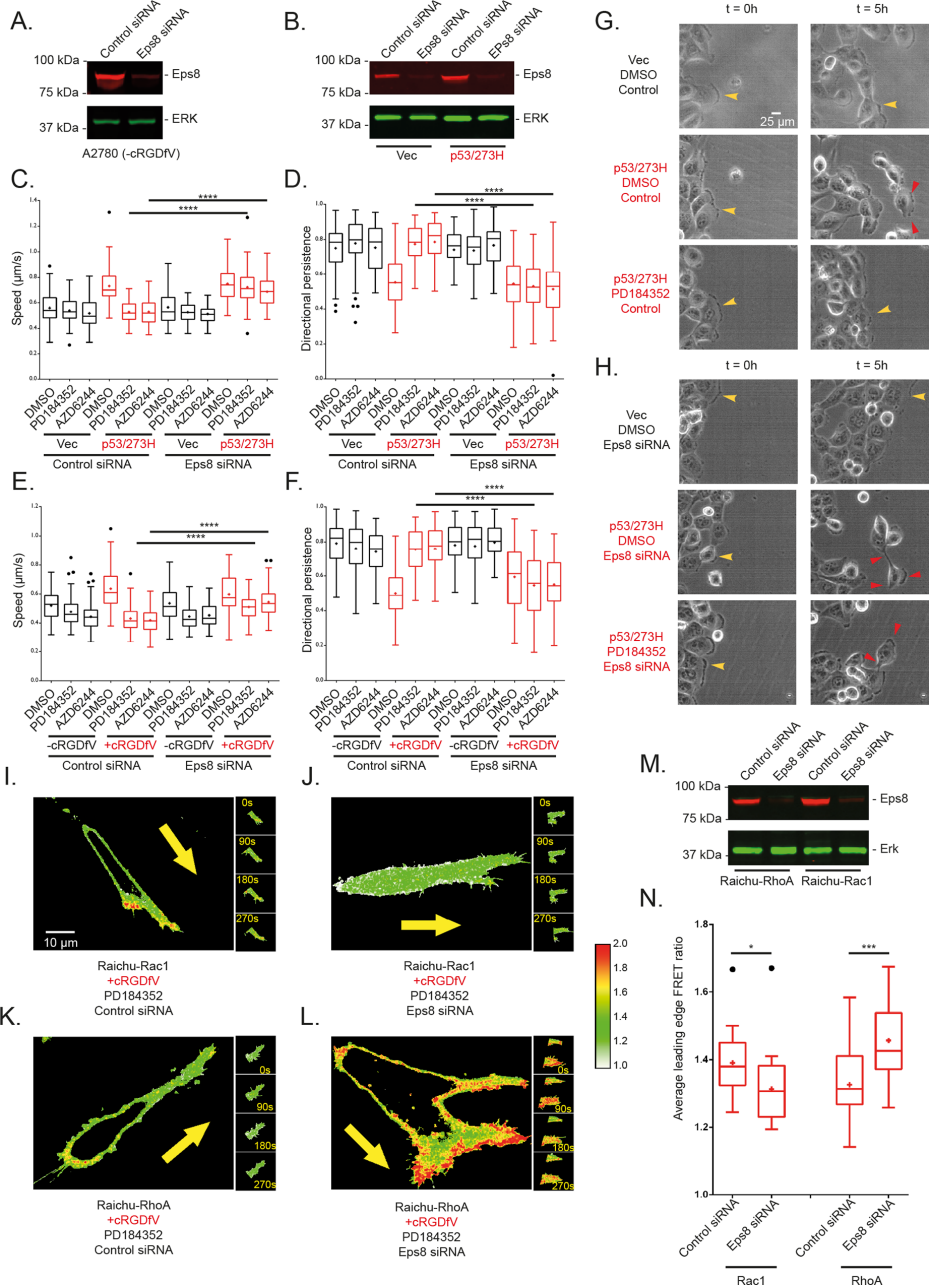
Eps8 siRNA renders cells insensitive to MEK1/2 inhibition. **A.** Western blot for total Eps8 levels and total Erk levels (for loading) in A2780 cells. Cells were subjected to a single nucleofection and lysed after 24 hours. The single nucleofection showed evident knockdown of Eps8 levels compared to control siRNA and were thus adopted throughout the rest of the study. **B.** Western blot for total Eps8 levels and total Erk levels (for loading) for H1299 cells stably expressing mutant p53 (right) or with an empty vector for mutant p53 (left). Both are single nucleofections with lysis 24 hours later and show evident knockdown of Eps8 compared to control siRNA. **C-H.** Effect of MEK1/2 inhibition and Eps8 knockdown on 2-D cell migration over >10 hours in scratch wound experiments. **C.** Average speed of tracked H1299 cells over the time taken for the wound to close or 16 hour duration of movies (whichever comes first). Cells stably expressing mutant p53 or with an empty vector for p53 were nucleofected with control siRNA or Eps8 siRNA, reseeded onto different culture dishes ~24 hours later and treated with DMSO/PD184352/AZD6244 ~ 1 hour prior to imaging. **D.** Average persistence for the same tracked H1299 cells as in C, persistence calculated as in 2G. **E.** Average speed of A2780 cells for the same conditions as in C, cells were stimulated with cRGDfV at the same time as treatment with DMSO/PD184352/AZD6244. **F.** Average persistence for the same tracked A2780 cells. In C-F, 3 experimental repeats were performed for each cell line with 30 cells tracked per condition. Graphs shown are Tukey boxplots with the mean represented as +; **** indicates p < 0.0001 in one way ANOVA with post hoc Tukey HSD test **G, H.** Representative images of H1299 cells in a sub-domain of an image at t = 0 (the initial frame) and the same sub-domain at t = 5 hours (30 10 minute frames later), for cells without/with mutant p53 expression, nucleofected with **G**. control siRNA or **H**. Eps8 siRNA and treated with DMSO or MEK1/2 inhibitor PD184352. Yellow arrow heads indicate lamellipodial leading edge actin, while red arrow heads indicate more spike-like protrusions. The same cell is highlighted with arrow heads at t = 0 and t = 5 hours for each different condition. **I-L.** Representative Ratiometric FRET images of whole A2780 cells on CDMs at single timepoints (main images) and leading edge quantified areas (panel of images, right). **I.** Cell nucleofected with Raichu-Rac1 probe and control siRNA (simultaneously) treated with cRGDfV and PD184352; **J.** Cell nucleofected with Raichu-Rac1 probe and Eps8 siRNA treated with cRGDfV and PD184352; **K.** Cell nucleofected with Raichu-RhoA probe and control siRNA treated with cRGDfV and PD184352; **L.** Cell nucleofected with Raichu-RhoA probe and Eps8 siRNA treated with cRGDfV and PD184352. As in 3E-H, All images have the same custom look-up table (LUT) applied and set between 1.0 and 2.0 (shown, right of images), where red pixels denote high GTPase activity. **M.** Western blot for total Eps8 levels and total Erk levels (for loading) in A2780 cells. Cells were nucleofected with control siRNA or Eps8 siRNA in the same solution as the Raichu Rac1 (right) or Raichu RhoA (left) probes and lysed ~24 hours later. Concomitant Raichu probe nucleofection had no effect on Eps8 knockdown as a clear knockdown was observed with either GTPase probe compared to control siRNA. **N.** Quantification of average FRET ratio in the leading edge of all analysed cells across all 20 timepoints in each 5 minute movie. N > 19 cells across 3 experimental repeats. Tukey boxplot used with mean indicated as +. Pairwise student t-tests used, * indicates p <0.05, *** indicates p <0.001.

Our Boolean model predicts that MEK-ERK signalling intervenes in the activation of Rac by influencing the activity of the Sos1 within the Sos1-Eps8-Abi1 complex [35,43]. Because MEK1/2 has been implicated in various cellular processes [46], we used knockdown of Eps8 as a method to remove the Rac-activating Sos1-Eps8-Abi1 complex in order to confirm the model prediction. One round of siRNA was sufficient to knock down Eps8 activity for both A2780 and H1299 cell lines using SMARTpool or single oligo siRNA reagents (Figs 4A and 4B and S5C). Eps8 siRNA had little effect on the lamellipodial migration of cells under basal conditions (Figs 4C–4F and 4H and S4A and S5A). RCP/α5β1/EGFR1 stimulated cell migration (A2780 +cRGDfV; H1299-mutant p53) in the absence of MEK1/2 inhibition was similarly unaffected by Eps8 knockdown, as a more rapid, random migratory phenotype remained. Whilst MEK inhibition was able to revert this rapid, random migratory phenotype in control knockdown cells (Figs 4C–4F and 4G and S4A and S5A, S3 Movie), Eps8 knockdown cells retained their fast migrating non-directional mode of movement in 2D even in the presence of MEK inhibitor (Figs 4C–4F and 4H and S4A and S5A, S3 Movie). These data provide further evidence that that a feedback loop initiated by MEK-ERK signalling acts to modulate migratory behaviour through a negative effect on the Sos1-Eps8-Abi1 complex, in agreement with the model prediction.

### The MEK feedback loop influences Rho GTPase signalling in an Eps8 dependent manner

To validate that the effect of Eps8 siRNA was indeed due to the downstream effects on Rac1 and RhoA, we again used FRET based biosensors to directly ascertain GTPase activity at the leading edge of cells migrating in 3D CDM. For control siRNA, the FRET ratios again showed that MEK1/2 inhibition promotes high Rac1 activity and low RhoA activity in lamellipodium-like protrusions (Figs 4I, 4K and 4N and S5B, S4 Movie). However Eps8 knockdown (Fig 4M) reduced Rac1 activity but concomitantly increased RhoA activity within the protrusive regions of migrating cells (Figs 4J, 4L and 4N and S5B, S4 Movie). These findings are again consistent with the model prediction that MEK1/2 inhibition affects localised Rac1 and RhoA activation dynamics via the Sos1-Eps8-Abi1, but has no effect when formation of this Rac1 activating complex is prevented by Eps8 siRNA.

### The MEK1/2-ERK feedback loop facilitates invasive migration downstream of RCP-dependent α5β1/EGFR1 co-trafficking and signalling

**Fig 5:**
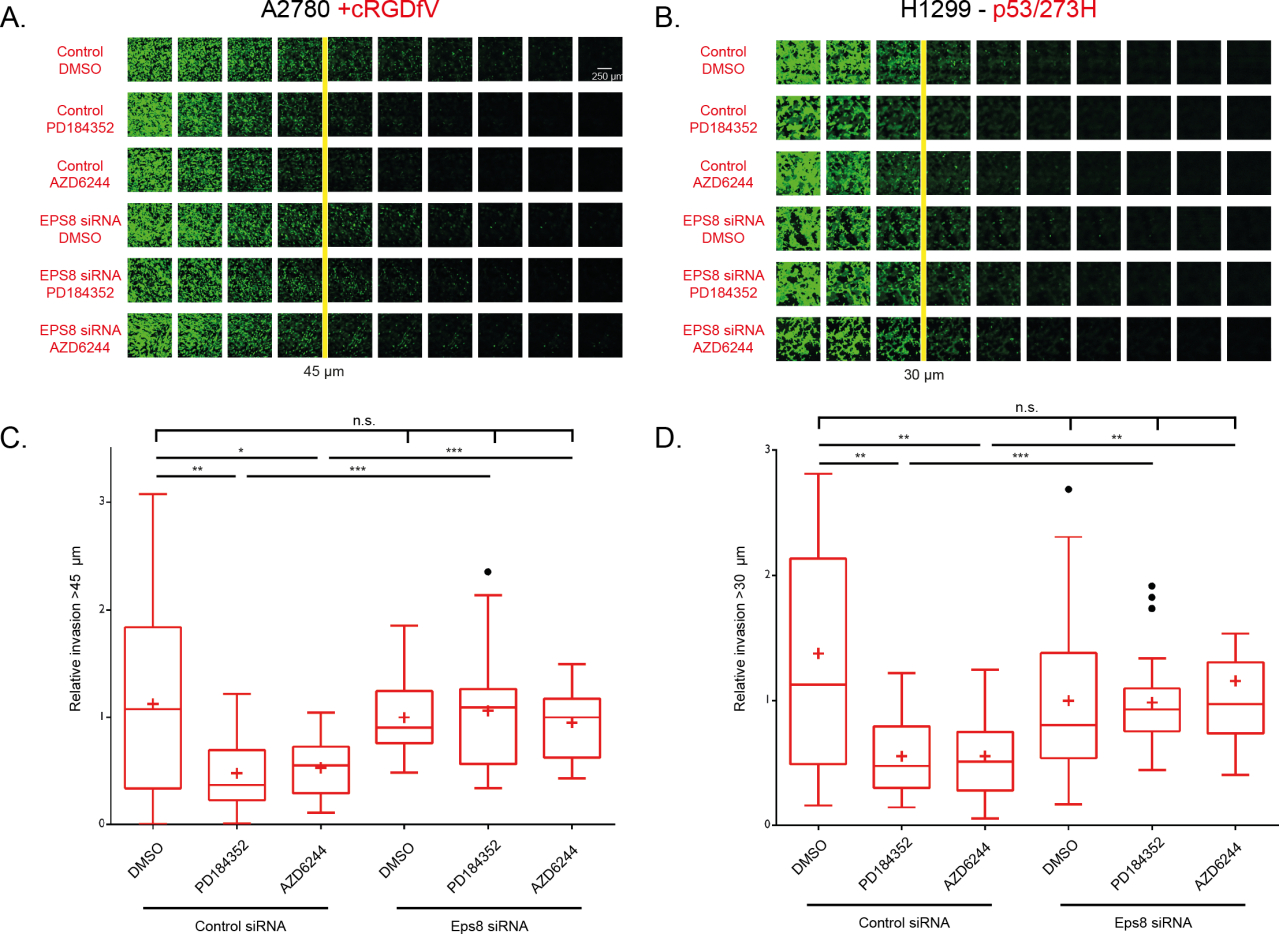
MEK1/2 inhibition significantly reduces α5β1/RCP driven 3-D invasion, Eps8 reverses the ME1/2 inhibition effect. **A-B.** Images of cells stained with calcein-AM following 48 hours of invasion into a fibronectin supplemented collagen matrix. Images shown are individual Z positions 14.97 μm apart, with the direction of invasion left to right for the images (the far left image for each condition is the bottom of the transwell). Cells were transfected with control siRNA or Eps8 siRNA 24 hours prior to seeding onto inverted transwells. MEK1/2 inhibitors or DMSO were used 24 hours after invasion was initiated to allow for proliferation. **A.** A2780 cells stimulated with cRGDfV throughout invasion assay with control/Eps8 siRNA and DMSO/PD184352 as indicated. 45 μm line has been taken as threshold for invasion, beyond which cells are thought of as invasive. **B.** As in A for H1299 cells stably expressing mutant p53. H1299 cells are less invasive so the 30 μm line has been taken as threshold for invasion. **C-D.** Quantification of at least 3-independent invasion assay experiments. Invasive proportion is calculated as the total GFP fluorescence for all the images above the invasive threshold (45 μm for A2780s, 30 μm for H1299s) divided by the total GFP fluorescence in all images. All data has been normalised to the Eps8 siRNA, DMSO treated condition to give relative invasion levels for all conditions for: **C.** A2780 cells stimulated with cRGDfV; **D.** H1299s cells stably expressing mutant p53. Graphs are Tukey boxplots with mean represented as +, individual student t-tests used as indicated, * indicates p <0.05, ** indicates p <0.01, *** indicates p < 0.001.

In cancer cells, the promotion of RCP-dependent α5β1/EGFR1 co-trafficking and signalling increases the invasive migration within FN-rich ECM hydrogels by mediating a Rac to RhoA switch at the leading edge to promote filopodial actin spike protrusions [20,21,23,24]. We therefore investigated whether the MEK1/2-ERK mediated feedback loop, which is required to maintain RhoA activity at the leading edge (Fig 3G–3I) influenced cell motility within 3D ECM that resembles interstitial matrix encountered by metastatic cancer cells. In the absence of Eps8 knockdown, MEK1/2 inhibition supressed the RCP-driven invasion of two different cell lines in high concentration collagen and FN hydrogels to <50% of uninhibited levels (Fig 5A–5D), consistent with previous observations [47] and providing more evidence to support MEK1/2 as a potential target to abrogate invasive cell migration. Strikingly, Eps8 knockdown A2780 and H1299 cells were unaffected by MEK1/2 inhibition, and showed a similar capacity to migrate and invade a 3D ECM when compared to untreated control knockdown cells (Fig 5A–5D), indicating that the Sos1-Eps8-Abi1 complex is critical MEK inhibition-induced phenotype reversion. These data demonstrate the regulation of the Rac to RhoA GTPase switch is critical for cancer cell invasion, and further support the model prediction that a Sos1-Ras-Raf-MEK-ERK negative feedback loop affects Rac1 and RhoA via the Sos1-Eps8-Abi1 complex to determine the mode of cell migration in 3D microenvironments.

## Discussion

We have used Boolean logic to model EGFR1 signalling at the front of the migrating cell in the context of RCP/ α5β1-mediated EGFR1 recycling, including over 40 nodes and interactions mined from the literature, which led to the prediction that a Sos1-Ras-Raf-MEK-ERK negative feedback loop affects localised Rac1 and RhoA dynamics at the leading edge of migrating cells via the Rac1 activating Sos1-Eps8-Abi1 complex. Using either of two MEK1/2 inhibitors with distinct mechanisms for suppression of target activity [44,45], we demonstrated that perturbing the MAP kinase pathway reverts the RCP/α5β1/EGFR1-driven rapid, random migration in 2D to a slower, more persistent, lamellipodial phenotype previously associated with dominant Rab4-αvβ3 integrin recycling [48]. In 3D-ECM, a similar reversion to lamellipodial protrusion was observed upon MEK inhibition, accompanied by increased Rac (but decreased RhoA) activity at the leading edge and a decrease in the ability to invade in FN-rich collagen hydrogels. Both the switching of GTPase activity and phenotypic reversion was dependent on Eps8, suggesting that the Sos1-Eps8-Abi1 complex is the target of the MEK-ERK driven feedback loop.

Our manually curated model was based on existing extensive EGFR networks found in the literature [32,49]. Proteins and interactions included in the final simulations are generally thought to be highly expressed and conserved across various different cell types, and proteins/interactions which resulted in biologically implausible results were rejected *a priori*. Based on experimental data, we accepted the Sos1E > pRacGAP1 > Vav2, RalBP1 Rac1 activator/inhibitor hierarchy. This particular hierarchy gave rise to cyclic activity of Rac1 and RhoA in a pseudo-oscillatory manner in the unperturbed simulation (Fig 1F). This was deemed plausible as it exhibited RhoA dominance at the leading edge of the cell downstream of RCP-α5β1 trafficking, as RhoA was ON for longer than Rac1. As a consequence of the negative feedback loop feeding into Sos1 activity however, cyclic activity was a natural consequence of this hierarchy. Using in silico node knockout workflow we identified a proposed mechanism through which the MAP kinase pathway affects GTPase activity and subsequent invasion. MEK1/2 is involved in a Sos1 negative feedback loop whereby cyclic activity of all the proteins in the loop is induced by ERK1/2 and/or p90RSK inhibiting Sos1 activity while also being activated downstream of Sos1 via a Ras-Raf-MEK cascade of activation. Following phosphorylation of Sos1 by ERK1/2 or p90RSK the activity of Sos1 has been shown to decrease, and the activity of other proteins involved in the MAP kinase pathway (including Ras) is also abrogated [43]. Sos1 has also been shown to activate Rac1 when in a complex with Eps8 and Abi1 [35,50,51], and Ras is thought to be involved in activating the Sos1-Eps8-Abi1 complex via PI3K [6,52]. This negative feedback loop may therefore affect the Sos1-Eps8-Abi1 indirectly via Ras as well as by directly downregulating Sos1 activity and availability in the leading edge (as explicitly modelled). In order to confirm the robustness of the model, and ensure that cyclic activity was not introduced as a consequence of the synchronous update algorithm used in our Boolean simulation, we used a random asynchronous updating scheme and confirmed that cyclic activity was observed in all simulations, and importantly RhoA activity remained dominant (S1A Fig). The model predicts that breaking the negative feedback loop activity via MEK1/2 removal leads to persistent (rather than cyclic) activity of the Sos1-Eps8-Abi1 complex which causes subsequent persistent activation of Rac1 and suppression of RhoA activity and thus a reduction in invasive cell migration. This feedback loop prediction was supported by the MEK1/2 effects as outlined above. Moreover, Eps8 knockdown via siRNA desensitises cells to MEK1/2 such that MAP kinase perturbation is no longer able to reduce cell migration speed or invasive capacity. FRET data indicates that these observed migration/invasion effects of Eps8 siRNA are also via leading edge Rac1 and RhoA dynamics, as predicted (Figs 4I–4N and S5B, S4 Movie).

Many studies have proposed possible roles for MEK1/2 in driving cell migration and invasion [53,54] via the Arp2/3 complex [55]. RCP-α5β1/EGFR1 trafficking and signalling promotes invasive cell migration in an Arp2/3-independent manner [24] and is thus a fundamentally different migratory system, yet it is interesting to note that in this context MEK inhibition promotes Rac activation and lamellipodia formation, presumable via the Arp2/3 complex. It is important to note that the effects driven downstream of RCP-mediated trafficking are localised specifically to the leading edge of cells because this is the location of vesicular trafficking [23,24], and hence global effects throughout the cell might not be expected. Previous studies have suggested that in cells harbouring K-RAS^G13D^ alleles, which lead to chronic stimulation of downstream signalling, ERK phosphorylates the RhoA GEF GEF-H1 and MEK inhibition in this context increases RhoA activity globally to promote actomyosin contractility and amoeboid migration [56]. In this study, we have employed cells lines that express wild-type K-Ras (A2780 ovarian cancer cells and H1299 non-small cell lung cancer cells [57,58]), and MEK inhibition leads to a localised decrease in RhoA activity, most likely due to a spatially restricted increase in Rac activation at the leading edge (Fig 3E–3I). We have previously shown that RCP-mediated α5β1/EGFR trafficking controls localised signalling [23] and hence it is likely that this apparent discrepancy is due to spatially restricted effects on cell signalling in cells that lack constitutive ERK activation.

Eps8 has been shown to have a role in actin filament capping [59] as well as in Rac1 activation when in complex with Sos1 and Abi1 [50]. Eps8 has been suggested to be an ERK effector, whereby both proteins must cooperate to drive migration via blebs [60]. Another study meanwhile has suggested that Eps8 regulates ERK activity which affects migration of breast cancer cells [61]. We find contrarily that MEK1/2 and Eps8 perturbation have opposite effects, whereby Eps8 siRNA desensitises cells to the anti-invasive effect of MEK1/2 inhibition. We propose that in our network, the main role of Eps8 is via its Rac1 activating capacity as part of the Sos1-Eps8-Abi1 complex as opposed to any effect on actin capping. Furthermore, knockdown of Eps8 has no effect on ERK activation in response to EGF (or MEK inhibitor induced suppression, S7 Fig).

Oscillation is an inherent part of the activation cycle of RhoGTPases at the single molecule level. Others have suggested that cyclic activity of RhoA and Rac1 is important in determining motility [13,14], and our model is also suggestive of this. However, direct evidence which supports pseudo-oscillatory Rac1 and RhoA activity is lacking at present. This is particularly due to current limitations in microscopy methods: i) wavelength constraints and the availability of suitable FRET pair fluorescent proteins dictate that only either Rac1 or RhoA activity, but not both, can be visualised in any single cell; ii) the channels required for FRET ratio calculations decree that GTPase activity can only be sampled at certain discrete timepoints (every 15 seconds in our experiments) making it difficult to reveal oscillations unless exactly in phase. This being said, FRET ratio results suggest that there is some fluctuation in both Rac1 and RhoA activity as cells migrate. Furthermore, there may be some cooperation of Rac1 and RhoA activity at the leading edge of motile cells as neither GTPase is completely ‘switched off’ (with a ratio of 1.0) or ‘switched on’ (with a ratio near 2.0). Therefore the ‘cyclic’ activity as simulated by the Boolean model is plausibly representative of experimental results–as all protein activity is binarised into ON or OFF statuses however, the only way to represent antagonistic proteins’ cooperation would be with a steady oscillation. It has previously been shown that negative circuits can generate cyclic output behaviour [62], and moreover it has been proven that negative circuits in a regulatory graph may be sufficient for such an observation [63]. These mathematical rules are observable in our model findings, whereby the RhoA dominant oscillations of Rac1 and RhoA are caused by the Sos1 negative feedback loop.

Our findings indicate that targeting MEK1/2 using specific drugs (such as Selumetinib—AZD6244 and PD184352 used here) may abrogate the harmful invasive migration and metastasis in certain contexts, particular for patients with tumours that harbour gain-of-function p53 mutation or which exhibit an upregulation in α5β1 expression. Targeting of the MAP kinase pathway has been ongoing in various clinical trials in thyroid, melanoma, ovarian and lung tumours due to the perceived effect that this greatly reduces tumour growth via decreased proliferation and increased apoptosis [64]. We propose MAP kinase targeting may additionally reduce metastasis to further benefit treatment of certain tumours.

Harnessing Boolean logic is a simple and effective method to study systems in an unbiased manner without the need for any parameters to be found or estimated. EGFR signalling activates a myriad of downstream effectors [65]. Moreover, the regulation of RhoA and Rac1 is a highly involved process involving the balance of GEFs, GAPs and their own regulators. A modelling approach based on known priors was consequently ideal to uncover further details regarding this pro-invasive signalling network. In particular, in silico workflows like those employed here could predict plausible therapeutic targets as well as derive further mechanistic insight. Such a modelling approach could be extended and adapted to pose specific questions regarding other growth factor signalling effects such as regarding proliferation or differentiation; alternatively, different GTPase networks or migratory events could also be recreated with Boolean logic.

Studying the signalling network in an integrated and connected manner as opposed to abstracting down to the minutia details of every molecular interaction permitted us to test complete unknowns with novel and unexpected experiments, including the Rac1 activator/inhibitor hierarchy and determining feedback via the Sos1-Eps8-Ab1 complex. With the advent of increasingly effective imaging techniques and ‘omics’ type large dataset derivation, more is understood *in vitro* about the complex signalling events at play in cancer cells. Mathematical approaches can complement the more traditional lab-based techniques, in particular to collate and rationalise wide-ranging data as well as to make novel and testable predictions to expand our present knowledge.

## Methods

### Curation of Boolean model

The Boolean model shown in Fig 1A was manually curated where all proteins and interactions were incorporated as follows. All proteins and interactions found to contribute to integrin-driven cell migration in [23] were first included in the model. Then several (>10) previously published Epidermal Growth Factor Receptor (EGFR) pathway maps were mined to find all proteins involved in interactions in pathways between (i) EGFR and Akt; and (ii) EGFR and Rac1/Cdc42 (note RhoA was not observed in any EGFR pathways), as EGFR, Akt and the Rho GTPases were key proteins implicated in [23]. In particular, the comprehensive pathway map produced by [65], which was subsequently adapted and formulated into a large scale logical model by [32], and the NetPath map by [49], contributed many proteins and interactions into the model. The model was then ‘cleaned’ by independent verification or rejection of proteins/interactions from studying the original papers cited in [32,49,65], rejecting interactions which give implausible output data following simulations, and omitting some proteins whose functions in the EGFR pathway are ambiguous and superfluous to model outputs. Reactions involving RhoA which were not included in [65] (such as activation by the known Rho/Rac GEF Vav2) were included based on literature evidence [31] and the necessity of full incorporation of RhoA.

### Boolean formulation

In the signalling network (Fig 1A), each node corresponds to a species or model variable (e.g. proteins in this case), and each directed edge corresponds to a different directed interaction between the nodes from and to which the edge is joining. Each node in the set of all nodes N is either in an active “ON” state or an inactive “OFF” state, denoted 1 and 0 respectively in a binary domain ∀ n ∊ N; t ∊ Z+: n(t) = 0 or 1. All reactions were simplified into activation or inhibition, and built using the 3 Boolean operators AND, OR, and NOT which are sufficient to represent any logical relationship. All reactions were assumed to take equal time, t = 1, then given the set of initial conditions in which at least one node is initially active, ∃ n ∊ N: n(0) = 1, nodes became active (transition from 0 → 1), inactive (1 → 0), or remained the same (0 →0 or 1→1) after each time increment depending on the state of upstream nodes and the interactions which the nodes were affected by. Where there was some ambiguity as to whether a reaction should involve “AND” or “OR” gates (particularly involving proteins found in [49] and not [32]) cited literature was studied to see if there was an explicit mention of two or more proteins acting together to affect a downstream node (in which case and “AND” gate was used) or not (in which case an “OR” gate was used).

### Boolean simulation and *in silico* knockouts

All Boolean simulations were performed in CellNetAnalyzer [66], a Matlab plugin. Reactions were coded according to the network graph in Fig 1A (except for reactions involving activation of Rac1 in which the role of RacGAP1 was important, see S3 Table) and then synchronous updates were performed for the first 50 time increments for all figures in the main text, giving rise to the full ON/OFF binary heat maps for all variables (Fig 1B and 1C). Where explicitly stated (S1A and S1B Fig discussion), random asynchronous updates were instead performed for the first 2000 time increments, whereby in any time increment at most one executable reaction will occur at random according to a uniform distribution. *In silico* knockouts and subsequent node classification was performed by removing all edges which lead into a single node and observing the effects on the output variables RhoA and Rac1. *In silico* knockouts were performed individually for all nodes in the model.

### Cell culture and transient transfections

A2780 cells were cultured in RPM1 1640, H1299 (expressing control vector or mutant p53-273H—[21]) and TIF cells were cultured in DMEM supplemented with 10% FCS and grown at 37°C and 5% CO_2_. Transient transfections of Raichu-RhoA and Raichu-Rac1 constructs and siRNA knockdowns of Eps8 were performed using the nucleofector (Solution T; 3 μg plasmid DNA or 1 μM siRNA; program A-23; Amaxa; Lonza), according to the manufacturer’s instructions. Both biosensor expression and Eps8 knockdown were obtained using one round of nucleofection, and Raichu-Rac1/RhoA and Eps8 siRNA were combined in the same nucleofection where required. All experiments were performed ~24 hours after nucleofection.

### Reagents

MEK inhibitors used were PD184352 [44] and AZD6244 [45], used at 1 μM in all experiments alongside DMSO as a control vehicle. Knockdown of Eps8 was performed using siRNA pools from an siARRAY (Dharmacon) and validated using two independent individual siRNAs: Eps8-A: 5’ GCGAGAGTCTATAGCCAAA; and Eps8-B: 5’ GCCAACTTCTAATCGCCATAT in comparison with a control siRNA. Biosensors used for FRET microscopy were kindly provided by Prof. M. Matsuda: Raichu-1011X (Rac1) [67] and Raichu-1237X (RhoA) [68]. Rabbit anti–phospho-p44/42 MAPK (ERK1/2) (Thr202/Tyr204) was purchased from Cell Signalling Technology, Rabbit anti–ERK2 (C-14) and Rabbit anti-Eps8 were purchased from Santa Cruz Biotechnology and Mouse anti–Eps8 was purchased from BD Biosciences. cRGDfV was purchased from BACHEM. Alexa Fluor 488 phalloidin used for actin staining was purchased from Life Technologies.

### SDS-PAGE and quantitative western blotting to test knockdown/inhibitor efficiency

Cells were treated exactly as in the conditions used for imaging experiments before being lysed in non-denaturing lysis buffer (200 mM NaCl, 75 mM Tris-HCl, pH 7.4, 15 mM NaF, 1.5 mM Na3VO4, 7.5 mM EDTA, 7.5 mM EGTA, 1.5% (v/v) Triton X-100, 0.75% (v/v) NP-40, 50 μg/ml leupeptin, 50 μg/ml aprotinin, and 1 mM 4-(2-aminoethyl)-benzenesulfonyl fluoride). Lysates were clarified by centrifugation at 10,000 g for 10 min at 4°C. Cell lysates were resolved under denaturing conditions by SDS-PAGE (4–12% Bis-Tris gels; Invitrogen) and transferred to nitrocellulose membrane. Membranes were blocked with 1x Blocking Buffer (Sigma) and incubated overnight at 4°C with the appropriate primary antibody in 5% BSA and then at room temperature for 1 h with the appropriate fluorophore-conjugated secondary antibody in 1x Blocking Buffer. Membranes were scanned using an infrared imaging system (Odyssey; LI-COR Biosciences). For quantification of western blots, mean intensity of each relevant band was measured using ImageJ. Loading was normalised to an appropriate loading control and backgrounds were subtracted. All conditions were normalised to the band with the highest intensity in each repeat, and then the mean normalised intensity was calculated across three independent repeats. Quantification of western blots was performed where explicitly stated.

### 2-D scratch-wound assay

Cells were grown to confluence and after 24 hours were scratched with a pipette tip before being washed once in appropriate serum containing medium and placed into fresh serum containing medium. Cells were treated with MEK inhibitors or DMSO at 1μM and cRGDfV at 2.5 μM as appropriate. Images were taken every 10 minutes for >84 time points (15 hours) for 6 different areas per condition. Cells were imaged using an AS MDW live cell imaging system (Leica) using a 20x/NA 0.50 Plan Fluotar Ph2 objective in brightfield. Micromanager imaging software and point visiting mode were used to allow multiple positions to be imaged within the same timecourse while cells were maintained at 37°C and 5% CO2. Images were collected using a Cascade II EM CCD camera (Photometrics). At least 5 cells per time-lapse position (giving 30 cells tracked per condition) were individually manually tracked using the ImageJ plugin MTrackJ [69] for the nucleus position every 3 frames (i.e. using 30 minute timepoint intervals). Cells were unbiasedly chosen and in general either started or ended at the leading edge of the scratch wound. The Chemotaxis and Migration Tool [70] was used to calculate the average speed and directional persistence for each given condition, where persistence is the ratio (displacement from initial position)/(total distance travelled).

### Inverted invasion assay

Inverted invasion assays were performed based on the protocol as described previously [71]. Collagen I (final concentration ∼5 μg/ml; BD Biosciences) supplemented with 25 μg/ml fibronectin was allowed to polymerise in inserts (Transwell; Corning) for 1 h at 37°C. Transwells were then inverted for cells to be seeded directly onto the underside at a concentration of 5 x10^5^ cells/ml for 2~4 hours at 37°C. Transwells were then re-inverted, washed twice in serum-free medium and placed in 0.1% serum medium treated with 2.5 μM cRGDfV (for A2780s). Medium supplemented with 10% FCS and 30 ng/ml EGF, treated with 2.5 μM cRGDfV was placed on top of the matrix, thereby providing a chemotactic gradient for invasion. After ~24 hours, the medium below the matrix was treated with 1 μM MEK inhibitor or DMSO as required, while the medium above the matrix was treated with 2 μM MEK inhibitor or DMSO. After a further ~24 hours, all cells were stained with Calcein-AM ~1 hour prior to imaging and visualised by confocal microscopy with serial optical sections being captured at 14.97-μm intervals using an inverted confocal microscope (TCS SP5 AOBS; Leica) using a 20× objective. Invasion was quantified using the area calculator plugin in ImageJ, where the invasive proportion was obtained by measuring the fluorescence intensity of cells invading > 45 μm for A2780s (the 5^th^ Z-stack onwards) and > 30 μm for H1299-273s (the 4^th^ Z-stack onwards), and dividing this by the total fluorescence intensity in all Z-stack images.

### FRET microscopy

All RhoA and Rac1 activity was determined by Forster resonance energy transfer (FRET) microscopy. Cells were transfected with RhoA or Rac1 probes and then seeded onto CDMs after ~18 hours at 5x10^4^ cells/ml and left for a further ~4 hours to spread appropriately for 3-D imaging. All growth medium was replaced by Ham’s F12 supplemented with 10% FCS and 25 mM Hepes buffer prior to imaging to achieve optimal visualising conditions and treated with 2.5 μM cRGDfV, 1 μM PD184352 or DMSO as required 1–2 hours pre-capture. Bio-sensor expressing cells were imaged every 15 seconds for the emission bands CFP (445nm, donor and acceptor), YFP (515nm donor and acceptor) and FRET (445nm CFP donor, 515nm YFP acceptor) with exposure times as follows in order: CFP– 800 ms, YFP– 400 ms, Fret– 800 ms for cells on CDMs. Images were acquired using a CSU-X1 spinning disc confocal (Yokagowa) on a Zeiss Axio-Observer Z1 microscope with a 63x/1.40 Plan-Apochromat objective, Evolve EMCCD camera (Photometrics) and motorised XYZ stage (ASI). The 445nm and 561nm lasers were controlled using an AOTF through the laserstack (Intelligent Imaging Innovations (3i)) allowing both rapid ‘shuttering’ of the laser and attenuation of the laser power. Images were captured using SlideBook 6.0 software (3i).

### FRET image processing and quantification

Using custom software written in Python and NumPy, the following semi-automated method was devised to quantify RhoA/Rac1 activity using ratio imaging (CFP donor—YFP acceptor channel over CFP donor—CFP acceptor channel) in a ring crescent region at the front of the cell: First and for each time points in the image stack, the channels were aligned to a 100th of a pixel by cross-correlation subpixel image registration. As the channel with the best signal to noise ratio, the YFP channel was used to create binary masks: the YFP donor—YFP acceptor Raichu probe stained images were then individually band-pass filtered (A trous wavelet, linear 3x3 filter, keeping scales 2–8) to remove both high frequency noise and stationary background. Images were thresholded using a fixed threshold (pixel grey value above 2000) and all the objects in the resulting binary image were identified by 8-connected component labelling. All but the largest object in the image were discarded, thus defining a time coded “cell mask” object for each image stack. Morphological operations (erosion, subtraction from the mask) were then combined to create a “ring mask” object, 40 pixels wide.

The axis aligned minimum bounding box of the “ring mask” objects was calculated and divided into four equal parts along the longer axis. The two extremal regions define two crescents of interest (front of the cell, back of the cell). Then at each subsequent time point, the newly calculated extremal regions were aligned to those found at the first time point. However manual user intervention was required to identify the actual front of the cell.

In order to calculate mean pixel ratios in this “front cell crescent” region of interest, the CFP donor—YFP acceptor and CFP donor—CFP acceptor images were smoothed using Gaussian Blur with a standard deviation of 1.0 pixel. This typically reduces wide pixel-to-pixel variations in the ratio image, with little effect on mean ratio values over larger areas. Only ratio image pixels with locations within the “front cell crescent” were calculated and averaged. We further avoided divisions by zero by ignoring pixels where the CFP donor—CFP acceptor values equal zero. Images shown are of the CFP-YFP/CFP-CFP ratio in the whole cell with a custom look-up table (LUT) set between values of 0.0 and 2.0.

For quantification, the leading edge FRET ratio was calculated as the average FRET ratio in the aforementioned masked area for each timepoint of every 20 frame, 5 minute movies. For comparison between conditions, the average of n = 15–20 cells across three independent repeats of the average FRET across every timepoint was chosen to give a single comparable Rac1/RhoA readout (S6 Fig).

### High-resolution imaging of actin structures

A2780 cells were seeded on CDMs at 5x10^4^ cells/ml and allowed to spread for >4 hours. Cells were then treated with cRGDfV/DMSO/PD184352 and left for a further >1 hour before being fixed in 4% paraformaldehyde and permeabilised with PBS + 0.2% Triton X. Cells were stained with Alexa Fluor 488 phalloidin over > 16 hours before being mounted in Prolong Gold antifade reagent.

Cells were imaged using a Leica TCS SP8 STED 3X microscope with an HC PL APO 100x/1.40 oil objective and further 3.0x or 4.0x confocal zoom. A pinhole with 0.7 Airy units was used to further improve resolution. Images were collected using a HyD detector with acceptance spectrum 504–595nm, 499nm laser line for emission. For whole cell images, one Z-plane was captured and images were not further deconvolved. For leading edge images, 14 Z-slice images were captured covering a total of Z distance of 2.99 μm. Deconvolution was then performed using Huygens Professional software with default settings. Images shown are maximum projections of deconvolved Z-stacks.

### Filopodia quantification

All quantifaction of filopodia was performed on deconvolved maximum projections of leading edge regions. Cells were cropped by a common sized rectangular ROI across different conditions and individually manually thresholded using ImageJ software such that pixels inside the cell (i.e. regions of high actin signal) were assigned a value >1 and regions outside the cell (background) were assigned a value of 0. Thresholded images were analysed using the Matlab application CellGeo [72] with a critical length = 20.0, critical width = 12.0 to automatically calculate number of filopodia per leading edge and average length of filopodia.

## Supporting Information

S1 Fig Random asynchronous model updates show similar output dynamics. **A.** Average RhoA and Rac1 node ON time following initial Rac1 OFF and RhoA ON switch during the first 2000 time increments: Asynchronous simulation data taken from 10 simulation repeats, Synchronous simulation data corresponds to deterministic heatmap in Fig 1C and has been scaled for direct comparison with Asynchronous data. **B.** Two typical heatmaps for random asynchronous simulations for the first 2000 time increments, all 10 simulations show similar cyclic RhoA/Rac1 activity.
(TIF)

S2 Fig Effects of MEK1/2 inhibition on 2-D A2780 cell migration in scratch wound experiments. **A.** Representative images of A2780 cells in a sub-domain of an image at t = 0 (the initial frame) and the same sub-domain at t = 5 hours (30 10 minute frames later), for cells without/with cRGDfV stimulation and treated with DMSO or MEK1/2 inhibitor AZD6244. Yellow arrow heads indicate lamellipodial leading edge actin, while red arrow heads indicate more spike-like protrusions. The same cell is highlighted with arrow heads at t = 0 and t = 5 hours for each different condition. Images correspond to quantified data in main Fig 2H and 2I.
(TIF)

S3 Fig MEK1/2 inhibition effect on Rac1 and RhoA activity of migrating H1299 cells expressing mutant p53. Representative Ratiometric FRET images of whole H1299-mutant p53 expressing cells on CDMs at a single timepoint. **A**. Cell transfected with Raichu-Rac1 probe, stimulated with cRGDfV and treated with DMSO for vehicle; **B.** Cell transfected with Raichu-Rac1 probe, stimulated with cRGDfV and treated with PD184352; **C**. Cell transfected with Raichu-RhoA probe, stimulated with cRGDfV and treated with DMSO for vehicle; **D**. Cell transfected with Raichu-RhoA probe, stimulated with cRGDfV and treated with PD184352. All images have the same custom look-up table (LUT) applied and set between 0.0 and 2.0 (shown, right of images), where red pixels denote high GTPase activity. **E.** Quantification of average FRET ratio in the leading edge of all analysed cells across all 20 timepoints in each 5 minute movie. N > 12 cells across 3 experimental repeats. Tukey boxplot used with mean indicated as +. Pairwise student t-tests used, * indicates p <0.05.
(TIF)

S4 Fig Effect of MEK1/2 inhibition and Eps8 knockdown on 2-D cell migration in scratch wound experiments. **A.** Representative images of A2780 cells in a sub-domain of an image at t = 0 (the initial frame) and the same sub-domain at t = 5 hours (30 10 minute frames later), for cells without/with cRGDfV stimulation, nucleofected with control siRNA or Eps8 siRNA and treated with DMSO or MEK1/2 inhibitor AZD6244. Yellow arrow heads indicate lamellipodial leading edge actin, while red arrow heads indicate more spike-like protrusions. The same cell is highlighted with arrow heads at t = 0 and t = 5 hours for each different condition. Images correspond to quantified data in main Fig 4E and 4F.
(TIF)

S5 Fig Individual Eps8 siRNA renders cells insensitive to MEK1/2 inhibition. **A.** Average speed and persistence of migrating A2780 cells into a scratch wound, treated exactly as in Fig 4C–4G, with either control siRNA, individual Eps8-A siRNA or individual Eps8-B siRNA as indicated. 3 experimental repeats were performed for each Eps8 siRNA experiment with >40 cells tracked per condition. Graphs shown are Tukey boxplots with the mean represented as +; **** indicates p < 0.0001 in one way ANOVA with post-hoc Tukey HSD test. **B.** Representative Ratiometric FRET images of whole A2780 cells treated with cRGDfV and PD184352, transfected with control siRNA, Eps8-A siRNA or Eps8-B siRNA as indicated reporting Rac1 activity (left) or RhoA activity (right). Graphs show average leading edge Fret activity calculated as in Fig 4N, >20 cells quantified for each Eps8 siRNA. Graphs shown are Tukey boxplots with the mean represented as +; ** indicates p < 0.01, *** indicates p < 0.001 and **** indicates p < 0.0001 in pairwise student t-tests. **C.** Western blots for total Eps8 (Rabbit anti-Eps8) levels and total Akt2 levels (for loading) in A2780 cells. Transfection efficiency was tested for 6 different individual siRNA oligos using the same single nucleofection and 24 hours later lysing conditions as with the smart pools in Fig 4A. The siRNAs labelled A and B above the bands showed the greatest knockdown versus the respective control siRNA and were thus used as indicated in the scratch wound and Fret assays in A and B.
(TIF)

S6 Fig Method for calculating average FRET ratio in the leading edge of migrating cells. **A.** Typical raw YFP-donor YFP-acceptor images at 4 different time points as of a migrating cell on a CDM nucleofected with the Raichu-RhoA probe (as well as Eps8 siRNA and treated with cRGDfV and PD184352). Images were taken with 400ms exposure. **B.** The same timepoints of the same cell following automatic thresholding (see Methods) where the YFP channel image has pixel values = 1.0 for pixels inside the cell (white) and = 0.0 for pixels outside the cell (black). **C.** The raw ratio images of CFP-donor YFP-acceptor images following the application of a Gaussian blur with ratio 1.0 pixel divided by the CFP-donor CFP-acceptor images following the application of a Gaussian blur with ratio 1.0 for the same cell timepoints as in B and C. **D.** FRET ratio images following removal of background noise. Binary mask images as in B are multiplied by raw ratio images as in C. The custom LUT is then applied to the images and set between 1.0 and 2.0 as in Figs 3E–3H and 4I–4L and S3A–S3D, S2 and S4 Movies where red pixels indicate high RhoA activity. **E.** Leading edge areas for all timepoints in the movie of the cell in A-D with the same LUT as in D applied. Leading edge is calculated following creation of a 40 pixel wide ring applied to the edge of the cell, and then the front 25% of this ring chosen along the long axis of the moving cell. **F.** Timecourse plot of the average leading edge FRET ratio for every timepoint of the moving cell as in E. Average FRET ratio is the mean average pixel intensity of all pixels within the masked leading edge region in E.
(TIF)

S7 Fig Eps8 siRNA has no direct effect on phosphorylated MAP kinase levels or MEK1/2 inhibition viability. **A.** Western blots showing total Eps8 levels, endogenous levels of phosphorylated p44 and p42 MAP Kinase (Erk1 and Erk2), and total levels of Akt2 (for loading) for A2780 cells either nucleofected with control siRNA or Eps8 siRNA and treated with PD184352 or DMSO for vehicle. **B**. Normalised quantification of p44 and p42 MAP Kinase intensity for cells either nucleofected with control siRNA or Eps8 siRNA and treated with PD184352 or DMSO for vehicle across three independent repeats. Error bars correspond to the standard error of the mean (SEM), individual pairwise student t-tests used.
(TIF)

S1 Movie H1299 cells migrating in a 2D scratch wound assay over > 16 hours, stably transfected with an empty vector for mutant p53 (left) or expressing mutant p53 (right) and treated with DMSO (top) or MEK1/2 inhibitor AZD6244 (bottom). (AVI)

S2 Movie FRET biosensors reporting Rac1 activity (top) or RhoA activity (bottom) for A2780 cells migrating over a 5 minute timecourse on 3D CDMs, stimulated with cRGDfV and treated with DMSO (left) or MEK1/2 inhibitor PD184352 (right). Thermal look up table applied as in Fig 3E–3H, where red denotes high GTPase activity and green denotes low GTPase activity.
(AVI)

S3 Movie H1299 cells migrating in a 2D scratch wound assay over > 10 hours, expressing mutant p53, treated with MEK1/2 inhibitor and transfected with control siRNA (left) or Eps8 siRNA (right) to abrogate formation of the Sos1-Eps8-Abi1 complex. (AVI)

S4 Movie FRET biosensors reporting Rac1 activity (top) or RhoA activity (bottom) for A2780 cells migrating over a 5 minute timecourse on 3D CDMs, stimulated with cRGDfV, treated with MEK1/2 inhibitor PD184352 and transfected with control siRNA (left) or Eps8 siRNA (right) to abrogate formation of the Sos1-Eps8-Abi1 complex. Thermal look up table applied as in Fig 4I–4L, where red denotes high GTPase activity and green denotes low GTPase activity.
(AVI)

S1 Table Every interaction in the model and references of origin. (DOCX)

S2 Table Initially active nodes in simulations and justification. All other nodes in the model are set to OFF and may become active at some time following EGF input.
(DOCX)

S3 Table Full list of Rac1 activator/inhibitor logical hierarchies. (DOCX)

S1 References Full list of references for information found in S1 and S2 Tables. (DOCX)

S1 Folder Contains all required code and instructions to simulate the model in CellNetAnalyzer (the MatLAB plugin). Raw code for reactions and species in the model can also be read by opening the ‘reactions’ or ‘metabolites’ file.
(ZIP)
