# Supplementary figures and images for: A MAPK-Driven Feedback Loop Suppresses Rac Activity to Promote RhoA-Driven Cancer Cell Invasion
Source: PLoS Comput Biol. 2016 May 3;12(5):e1004909. doi: 10.1371/journal.pcbi.1004909 (PMC4854413; doi:10.1371/journal.pcbi.1004909)

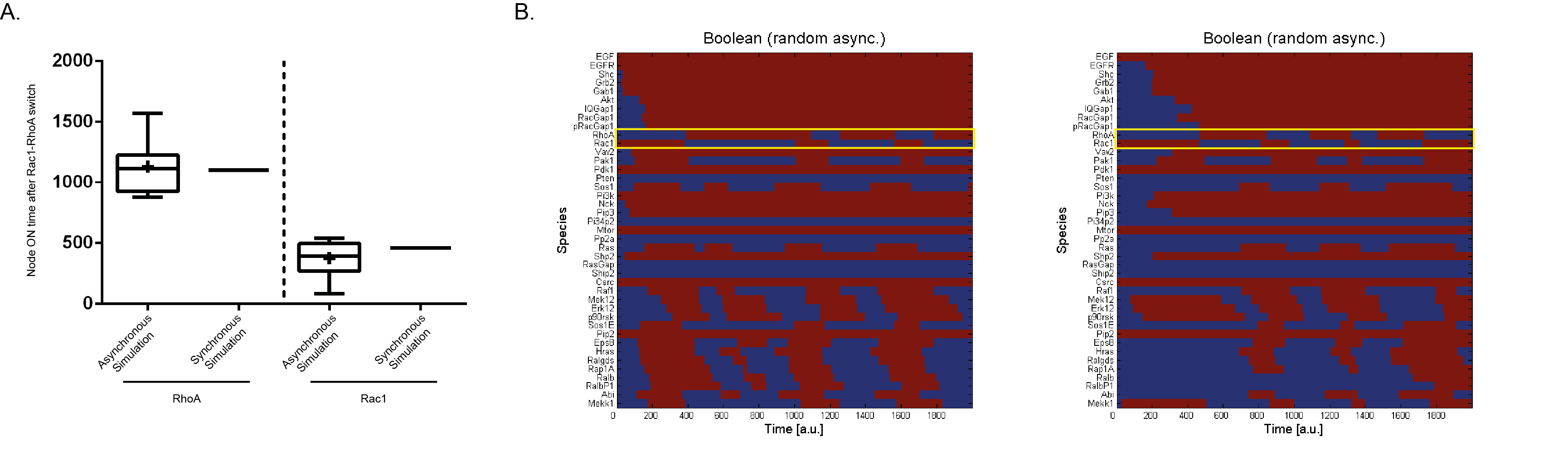

Supplement: S1 Fig — A. Average RhoA and Rac1 node ON time following initial Rac1 OFF and RhoA ON switch during the first 2000 time increments: Asynchronous simulation data taken from 10 simulation repeats, Synchronous simulation data corresponds to deterministic heatmap in Fig 1C and has been scaled for direct comparison with Asynchronous data. B. Two typical heatmaps for random asynchronous simulations for the first 2000 time increments, all 10 simulations show similar cyclic RhoA/Rac1 activity. (TIF) [file pcbi.1004909.s001.tif]

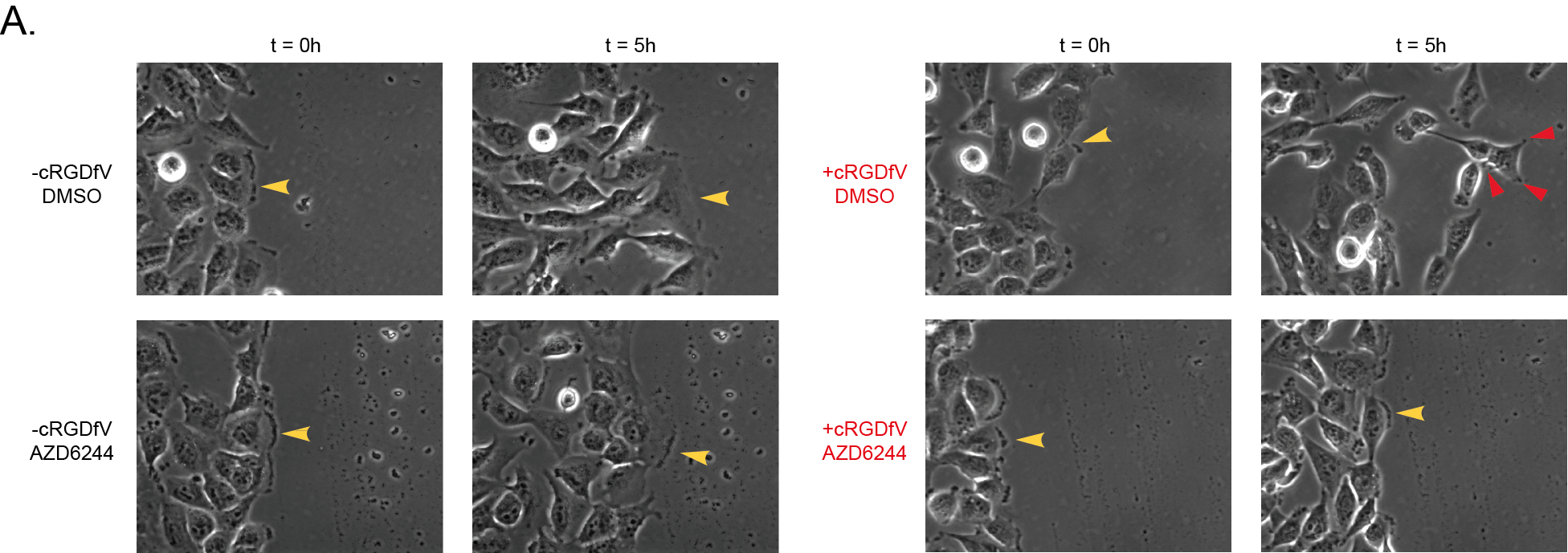

Supplement: S2 Fig — A. Representative images of A2780 cells in a sub-domain of an image at t = 0 (the initial frame) and the same sub-domain at t = 5 hours (30 10 minute frames later), for cells without/with cRGDfV stimulation and treated with DMSO or MEK1/2 inhibitor AZD6244. Yellow arrow heads indicate lamellipodial leading edge actin, while red arrow heads indicate more spike-like protrusions. The same cell is highlighted with arrow heads at t = 0 and t = 5 hours for each different condition. Images correspond to quantified data in main Fig 2H and 2I. (TIF) [file pcbi.1004909.s002.tif]

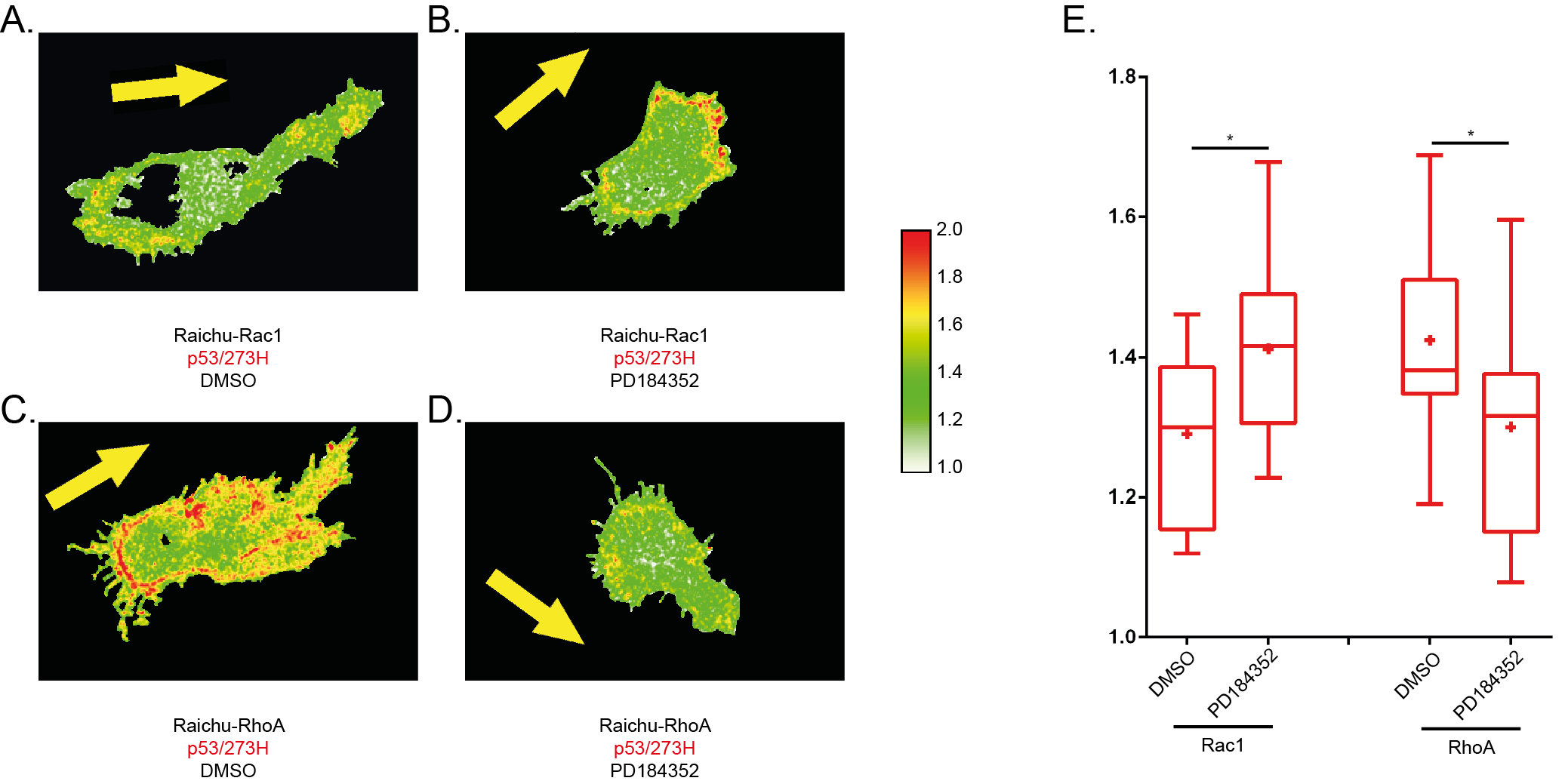

Supplement: S3 Fig — Representative Ratiometric FRET images of whole H1299-mutant p53 expressing cells on CDMs at a single timepoint. A. Cell transfected with Raichu-Rac1 probe, stimulated with cRGDfV and treated with DMSO for vehicle; B. Cell transfected with Raichu-Rac1 probe, stimulated with cRGDfV and treated with PD184352; C. Cell transfected with Raichu-RhoA probe, stimulated with cRGDfV and treated with DMSO for vehicle; D. Cell transfected with Raichu-RhoA probe, stimulated with cRGDfV and treated with PD184352. All images have the same custom look-up table (LUT) applied and set between 0.0 and 2.0 (shown, right of images), where red pixels denote high GTPase activity. E. Quantification of average FRET ratio in the leading edge of all analysed cells across all 20 timepoints in each 5 minute movie. N > 12 cells across 3 experimental repeats. Tukey boxplot used with mean indicated as +. Pairwise student t-tests used, * indicates p <0.05. (TIF) [file pcbi.1004909.s003.tif]

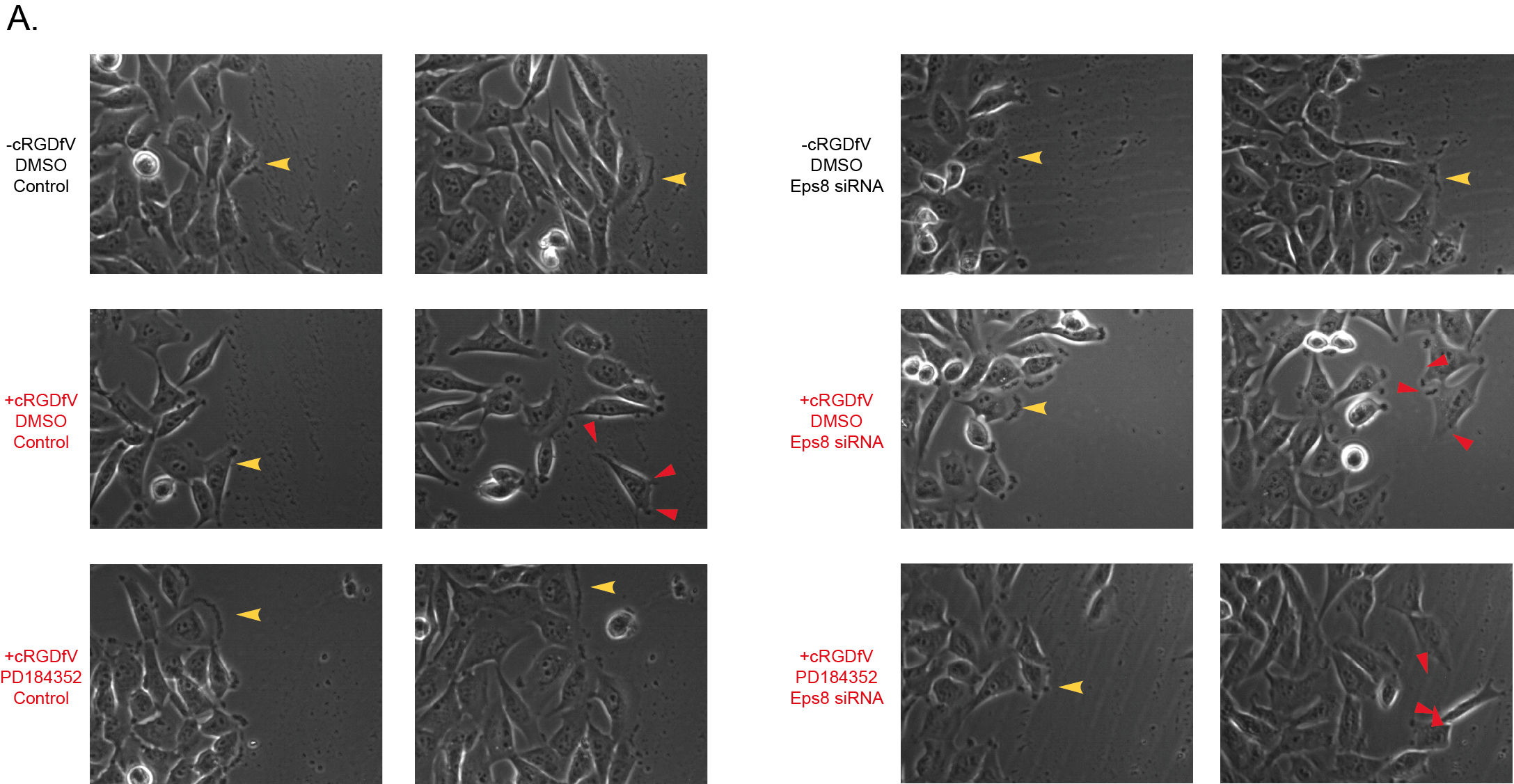

Supplement: S4 Fig — A. Representative images of A2780 cells in a sub-domain of an image at t = 0 (the initial frame) and the same sub-domain at t = 5 hours (30 10 minute frames later), for cells without/with cRGDfV stimulation, nucleofected with control siRNA or Eps8 siRNA and treated with DMSO or MEK1/2 inhibitor AZD6244. Yellow arrow heads indicate lamellipodial leading edge actin, while red arrow heads indicate more spike-like protrusions. The same cell is highlighted with arrow heads at t = 0 and t = 5 hours for each different condition. Images correspond to quantified data in main Fig 4E and 4F. (TIF) [file pcbi.1004909.s004.tif]

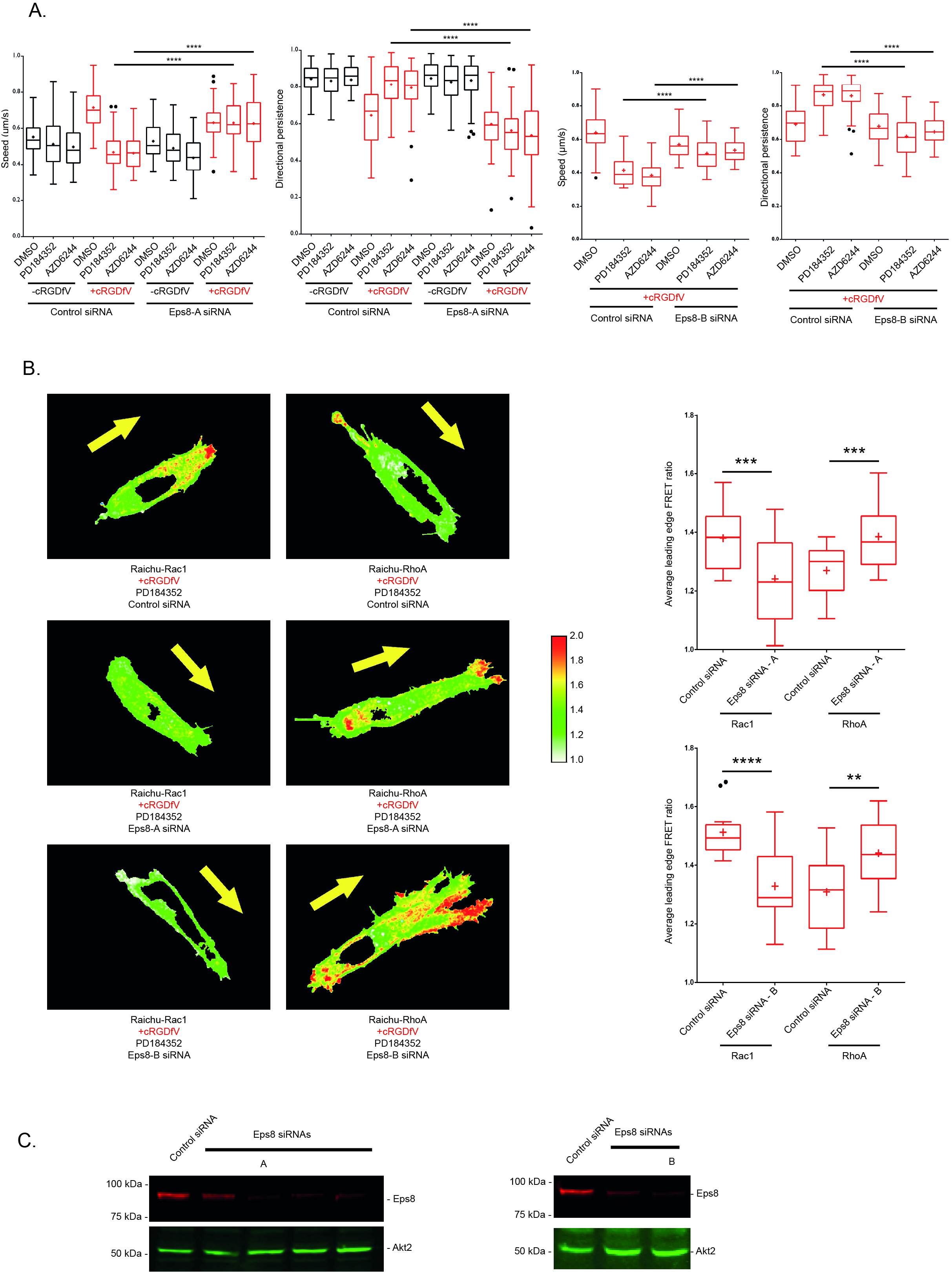

Supplement: S5 Fig — A. Average speed and persistence of migrating A2780 cells into a scratch wound, treated exactly as in Fig 4C–4G, with either control siRNA, individual Eps8-A siRNA or individual Eps8-B siRNA as indicated. 3 experimental repeats were performed for each Eps8 siRNA experiment with >40 cells tracked per condition. Graphs shown are Tukey boxplots with the mean represented as +; **** indicates p < 0.0001 in one way ANOVA with post-hoc Tukey HSD test. B. Representative Ratiometric FRET images of whole A2780 cells treated with cRGDfV and PD184352, transfected with control siRNA, Eps8-A siRNA or Eps8-B siRNA as indicated reporting Rac1 activity (left) or RhoA activity (right). Graphs show average leading edge Fret activity calculated as in Fig 4N, >20 cells quantified for each Eps8 siRNA. Graphs shown are Tukey boxplots with the mean represented as +; ** indicates p < 0.01, *** indicates p < 0.001 and **** indicates p < 0.0001 in pairwise student t-tests. C. Western blots for total Eps8 (Rabbit anti-Eps8) levels and total Akt2 levels (for loading) in A2780 cells. Transfection efficiency was tested for 6 different individual siRNA oligos using the same single nucleofection and 24 hours later lysing conditions as with the smart pools in Fig 4A. The siRNAs labelled A and B above the bands showed the greatest knockdown versus the respective control siRNA and were thus used as indicated in the scratch wound and Fret assays in A and B. (TIF) [file pcbi.1004909.s005.tif]

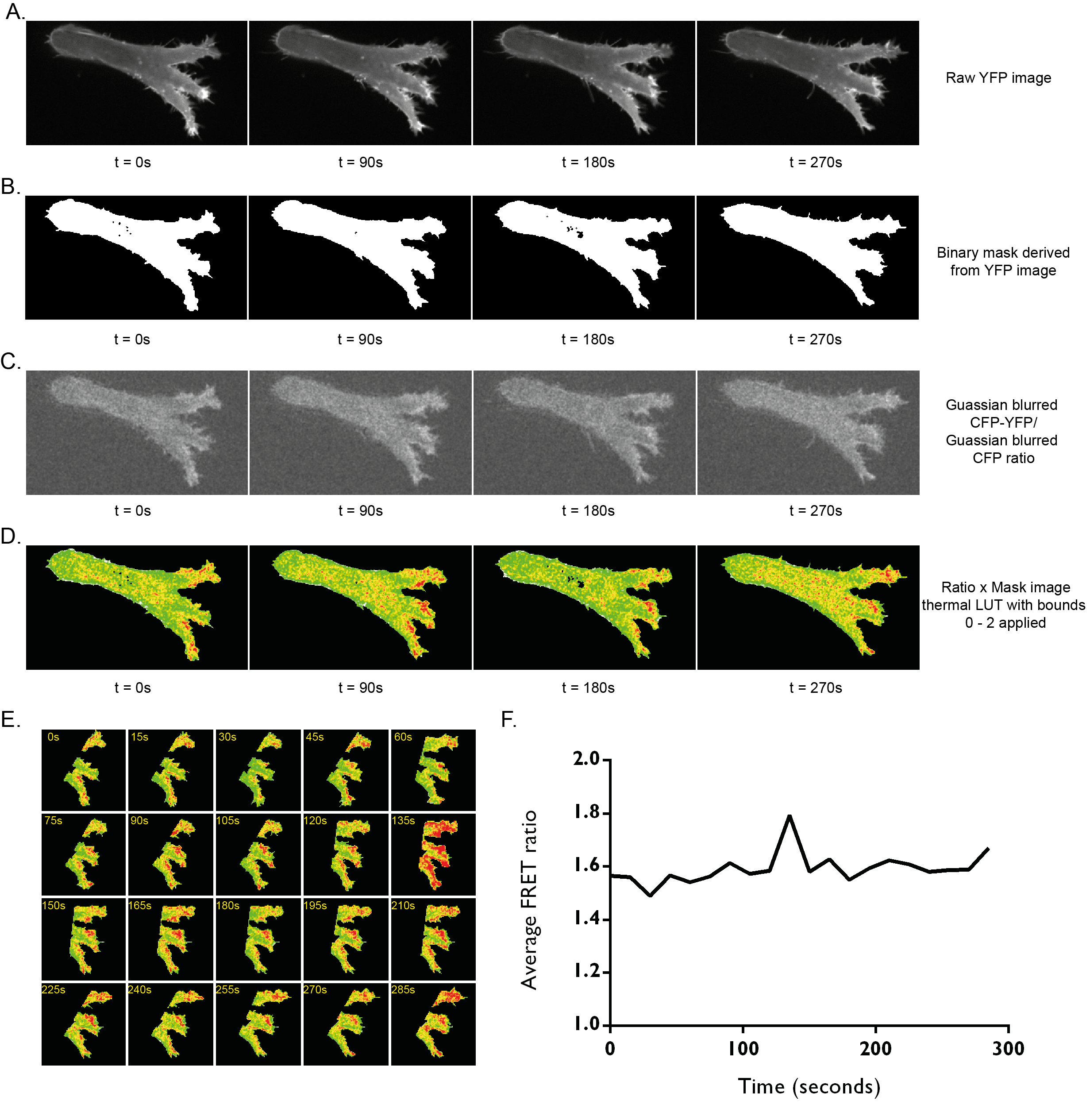

Supplement: S6 Fig — A. Typical raw YFP-donor YFP-acceptor images at 4 different time points as of a migrating cell on a CDM nucleofected with the Raichu-RhoA probe (as well as Eps8 siRNA and treated with cRGDfV and PD184352). Images were taken with 400ms exposure. B. The same timepoints of the same cell following automatic thresholding (see Methods) where the YFP channel image has pixel values = 1.0 for pixels inside the cell (white) and = 0.0 for pixels outside the cell (black). C. The raw ratio images of CFP-donor YFP-acceptor images following the application of a Gaussian blur with ratio 1.0 pixel divided by the CFP-donor CFP-acceptor images following the application of a Gaussian blur with ratio 1.0 for the same cell timepoints as in B and C. D. FRET ratio images following removal of background noise. Binary mask images as in B are multiplied by raw ratio images as in C. The custom LUT is then applied to the images and set between 1.0 and 2.0 as in Figs 3E–3H and 4I–4L and S3A–S3D, S2 and S4 Movies where red pixels indicate high RhoA activity. E. Leading edge areas for all timepoints in the movie of the cell in A-D with the same LUT as in D applied. Leading edge is calculated following creation of a 40 pixel wide ring applied to the edge of the cell, and then the front 25% of this ring chosen along the long axis of the moving cell. F. Timecourse plot of the average leading edge FRET ratio for every timepoint of the moving cell as in E. Average FRET ratio is the mean average pixel intensity of all pixels within the masked leading edge region in E. (TIF) [file pcbi.1004909.s006.tif]

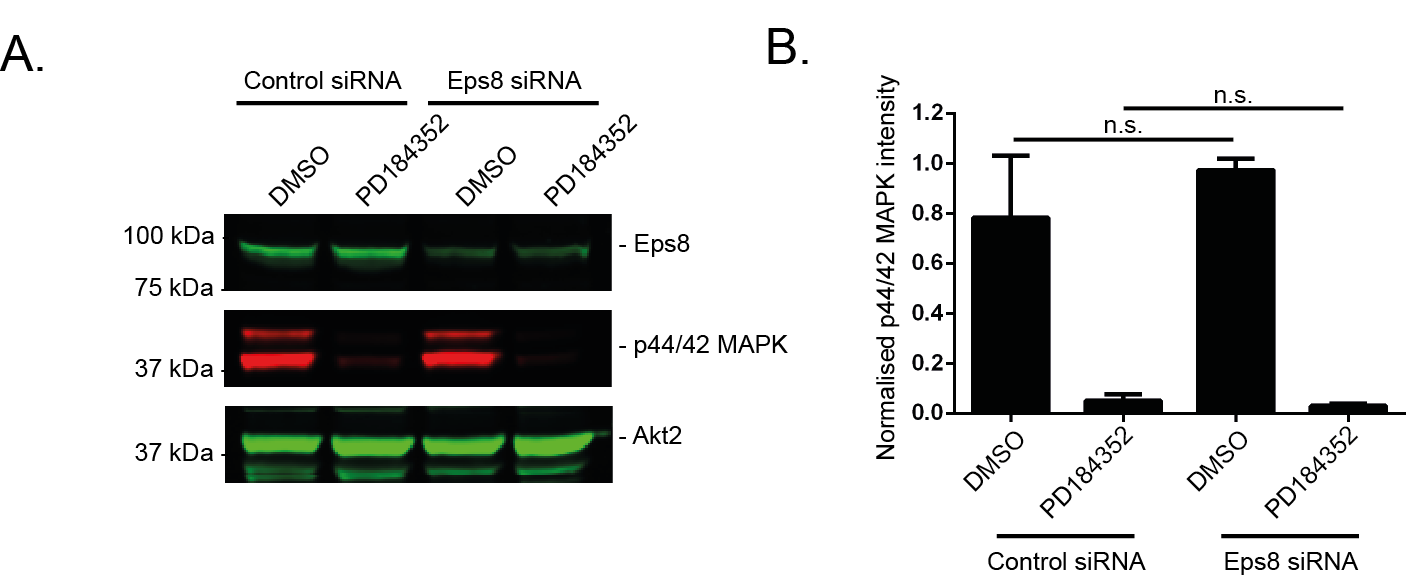

Supplement: S7 Fig — A. Western blots showing total Eps8 levels, endogenous levels of phosphorylated p44 and p42 MAP Kinase (Erk1 and Erk2), and total levels of Akt2 (for loading) for A2780 cells either nucleofected with control siRNA or Eps8 siRNA and treated with PD184352 or DMSO for vehicle. B. Normalised quantification of p44 and p42 MAP Kinase intensity for cells either nucleofected with control siRNA or Eps8 siRNA and treated with PD184352 or DMSO for vehicle across three independent repeats. Error bars correspond to the standard error of the mean (SEM), individual pairwise student t-tests used. (TIF) [file pcbi.1004909.s007.tif]

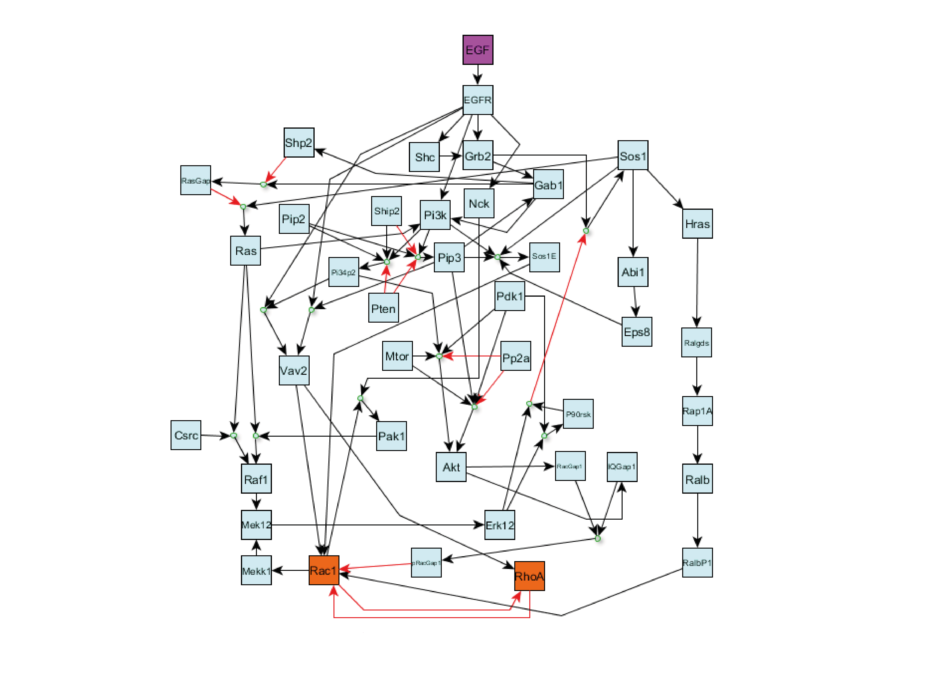

Supplement: S1 Folder — Raw code for reactions and species in the model can also be read by opening the ‘reactions’ or ‘metabolites’ file. (ZIP) [file pcbi.1004909.s016.zip › Hetmanski_model/network map.bmp]
