# Supplementary material for: A MAPK-Driven Feedback Loop Suppresses Rac Activity to Promote RhoA-Driven Cancer Cell Invasion
Source: PLoS Comput Biol. 2016 May 3;12(5):e1004909. doi: 10.1371/journal.pcbi.1004909 (PMC4854413; doi:10.1371/journal.pcbi.1004909)
Supplement: S1 Table — (DOCX) [file pcbi.1004909.s012.docx]

| **Interaction** | **Reference** |
| --- | --- |
| EGF → EGFR | Cohen, 1986 |
| EGFR → Shc | Olayioye et al., 2000 |
| EGFR → Grb2 | Schulze et al., 2005 |
| EGFR → Nck | Li et al., 2001 |
| EGFR → Pi3k | Olayioye et al., 2000 |
| Shc → Grb2 | Okabayashi et al., 1994 |
| Grb2 → Gab1 | Rodrigues et al., 2000 |
| Gab1 → Pi3k | Montagner et al., 2005 |
| Pi3k + Pip2 + !Ship2 + !Pten → Pip3 | Vanhaesebroeck et al., 1997; Scheid & Woodgett 2003 |
| Pi3k + Pip2 + Ship2 + !Pten → Pi34p2 | Vanhaesebroeck et al., 1997; Scheid & Woodgett 2003 |
| Pip3 → Gab1 | Rodrigues et al., 2000 |
| Pip3 + Pdk1 + Mtor + !Pp2a → Akt | Sarbassov et al., 2005a; Scheid & Woodgett 2003; Andjelkovic et al., 1996 |
| Pi34p2 + Pdk1 + Mtor + !Pp2a → Akt | Sarbassov et al., 2005a; Scheid & Woodgett 2003; Andjelkovic et al., 1996 |
| Akt → RacGap1 | Jacquemet et al., 2013 |
| Akt → IQGap1 | Jacquemet et al., 2013 |
| RacGap1 + IQGap1 → pRacGap1 | Jacquemet et al., 2013 |
| !pRacGap1 → Rac1 | Toure et al., 1998 |
| EGFR + Pip3 → Vav2 | Tamas et al., 2003 |
| EGFR + Pi34p2 → Vav2 | Tamas et al., 2003 |
| Vav2 → Rac1 | Tamas et al., 2003 |
| Vav2 → RhoA | Patel & Karginov, 2013 |
| Gab1 → Shp2 | Montagner et al., 2005 |
| Gab1 + !Shp2 → RasGap | Montagner et al., 2005 |
| Sos1 + !RasGap → Ras | Li et al., 1993; Cox & Der, 2003 |
| Ras → Pi3k | Downward, 1998 |
| Ras + Csrc → Raf1 | King et al., 2001 |
| Ras + Pak1 → Raf1 | King et al., 2001 |
| Raf1 → Mek12 | Chen et al., 2001 |
| Rac1 + Nck → Pak1 | Edwards et al., 1999 |
| Rac1 → Mekk1 | Schlesinger et al., 1998 |
| Mekk1 → Mek12 | Schlesinger et al., 1998 |
| Mek12 → Erk12 | Robinson & Cobb, 1997 |
| Erk12 + Pdk1 → P90rsk | Frodin et al., 2000 |
| Grb2 + !Erk12 + !P90rsk → Sos1 | Douville & Downward., 1997 |
| Sos1 → Abi1 | Okada et al., 1996; Scita et al., 1999 |
| Abi1 → Eps8 | Scita et al., 1999 |
| Sos1 + Pip3 + Pi3k + Eps8 → Sos1E | Innocenti et al., 2003 |
| Sos1E → Rac1 | Innocenti et al., 2003 |
| Sos1 → Hras | Margarit et al., 2003 |
| Hras → Ralgds | Kikuchi & Williams., 1996 |
| Ralgds → Rap1A | Matsubara et al., 1999 |
| Rap1A → Ralb | Nancy et al., 1999 |
| Ralb → RalbP1 | Ikechi et al., 1998 |
| RalbP1 → Rac1 | Kandasamy et al., 2010; Wang et al., 2013 |
| !Rac1 → RhoA | Iden & Collard., 2008 |
| !RhoA → Rac1 | Iden & Collard., 2008 |
