# Supplementary material for: A MAPK-Driven Feedback Loop Suppresses Rac Activity to Promote RhoA-Driven Cancer Cell Invasion
Source: PLoS Comput Biol. 2016 May 3;12(5):e1004909. doi: 10.1371/journal.pcbi.1004909 (PMC4854413; doi:10.1371/journal.pcbi.1004909)
Supplement: S2 Table — All other nodes in the model are set to OFF and may become active at some time following EGF input. (DOCX) [file pcbi.1004909.s013.docx]

| **Node** | **Justification** | **Reference** |
| --- | --- | --- |
| EGF | Input of model, Extracellular EGF is required to initiate the signalling network. | N/A |
| Pdk1 | Pdk1 appears to be constitutively active. | Newton, 2003 |
| Mtor | Regulation of Mtor is unknown; however it has been previously assumed that Mtor can be activated by its own resevoir. Akt has been observed to be activated downstream of EGF-EGFR binding and Mtor must be active for this to occur. | Sarbassov et al., 2005b; Samaga et al., 2009; Caswell et al., 2008 |
| Csrc | Src is activated by various mechanisms aside from receptor signalling, notably adhesion receptors such as integrins. Therefore Csrc may be assumed to be actived by unmodelled adhesion receptor properties not explicitly modelled. | Arias-Salgado et al., 2003 |
| Pip2 | Pip2 is the major phosphorylated form of PtdIns so is assumed to always be present in the cell. Pip2 is also required for the observed Akt activation via Pip3. | Tolias & Cantley, 1999 |
| Rac1 | Basal case of normally non-invasive motile cells show high Rac1 activity at the leading edge without cotrafficking of EGFR with α5β1 and RCP, hence Rac1 taken as ON before signalling pathway is initiated by EGF-EGFR binding. | Caswell et al., 2008 |
