# Supplementary material for: A MAPK-Driven Feedback Loop Suppresses Rac Activity to Promote RhoA-Driven Cancer Cell Invasion
Source: PLoS Comput Biol. 2016 May 3;12(5):e1004909. doi: 10.1371/journal.pcbi.1004909 (PMC4854413; doi:10.1371/journal.pcbi.1004909)
Supplement: S3 Table — (DOCX) [file pcbi.1004909.s014.docx]

| **Hierarchy** | **Corresponding Boolean equations** | **Rac1/RhoA Output** |
| --- | --- | --- |
| Vav2, RalbP1, Sos1E > pRacGAP1 | Vav2 → Rac1 | Rac1 remains ON |
|  | RalbP1 → Rac1 |  |
|  | Sos1E → Rac1 |  |
|  | !pRacGAP1 → Rac1 |  |
| Vav2, RalbP1 > pRacGAP1 > Sos1E | Vav2 → Rac1 | Rac1 remains ON |
|  | RalbP1 → Rac1 |  |
|  | Sos1E + !pRacGAP1→ Rac1 |  |
|  | !pRacGAP1 → Rac1 |  |
| Vav2, Sos1E > pRacGAP1 > RalbP1 | Vav2 → Rac1 | Rac1 remains ON |
|  | RalbP1 + !pRacGAP1 → Rac1 |  |
|  | Sos1E → Rac1 |  |
|  | !pRacGAP1 → Rac1 |  |
| Vav2 > pRacGAP1 > RalbP1, Sos1E | Vav2 → Rac1 | Rac1 remains ON |
|  | RalbP1 + !pRacGAP1→ Rac1 |  |
|  | Sos1E + !pRacGAP1→ Rac1 |  |
|  | !pRacGAP1 → Rac1 |  |
| RalbP1, Sos1E > pRacGAP1 > Vav2 | Vav2 + !pRacGAP1 → Rac1 | cyclic RhoA/Rac1 activity |
|  | RalbP1 → Rac1 |  |
|  | Sos1E → Rac1 |  |
|  | !pRacGAP1 → Rac1 |  |
| RalbP1 > pRacGAP1 > Vav2, Sos1E | Vav2 + !pRacGAP1 → Rac1 | cyclic RhoA/Rac1 activity |
|  | RalbP1 → Rac1 |  |
|  | Sos1E + !pRacGAP1→ Rac1 |  |
|  | !pRacGAP1 → Rac1 |  |
| Sos1E > pRacGAP1 > Vav2, RalbP1 | Vav2 + !pRacGAP1 → Rac1 | RhoA dominant cyclic  RhoA/Rac1 activity |
|  | RalbP1 + !pRacGAP1→ Rac1 |  |
|  | Sos1E → Rac1 |  |
|  | !pRacGAP1 → Rac1 |  |
| pRacGAP1 > Vav2, RalbP1, Sos1E | Vav2 + !pRacGAP1 → Rac1 | Rac1 to RhoA switch |
|  | RalbP1 + !pRacGAP1→ Rac1 |  |
|  | Sos1E + !pRacGAP1→ Rac1 |  |
|  | !pRacGAP1 → Rac1 |  |
