# Supplementary material for: A MAPK-Driven Feedback Loop Suppresses Rac Activity to Promote RhoA-Driven Cancer Cell Invasion
Source: PLoS Comput Biol. 2016 May 3;12(5):e1004909. doi: 10.1371/journal.pcbi.1004909 (PMC4854413; doi:10.1371/journal.pcbi.1004909)
Supplement: S1 References — (DOCX) [file pcbi.1004909.s015.docx]

Supplementary References

Andjelković, N., Zolnierowicz, S., Van Hoof, C., Goris, J., & Hemmings, B. A. (1996). The catalytic subunit of protein phosphatase 2A associates with the translation termination factor eRF1. The EMBO journal, 15(24), 7156.

Arias-Salgado, E. G., Lizano, S., Sarkar, S., Brugge, J. S., Ginsberg, M. H., & Shattil, S. J. (2003). Src kinase activation by direct interaction with the integrin β cytoplasmic domain. Proceedings of the National Academy of Sciences, 100(23), 13298-13302.

Chen, J., Fujii, K., Zhang, L., Roberts, T., & Fu, H. (2001). Raf-1 promotes cell survival by antagonizing apoptosis signal-regulating kinase 1 through a MEK–ERK independent mechanism. Proceedings of the National Academy of Sciences, 98(14), 7783-7788.

Cohen, S. (1986). Epidermal growth factor. Bioscience reports, 6(12), 1017-1028.

Cox, A. D., & Der, C. J. (2003). The dark side of Ras: regulation of apoptosis. Oncogene, 22(56), 8999-9006.

Douville, E., & Downward, J. (1997). EGF induced SOS phosphorylation in PC12 cells involves P90 RSK-2. Oncogene, 15(4), 373-383.

Downward, J. (1998). Ras signalling and apoptosis. Current opinion in genetics & development, 8(1), 49-54.

Edwards, D. C., Sanders, L. C., Bokoch, G. M., & Gill, G. N. (1999). Activation of LIM-kinase by Pak1 couples Rac/Cdc42 GTPase signalling to actin cytoskeletal dynamics. Nature cell biology, 1(5), 253-259.

Frödin, M., Jensen, C. J., Merienne, K., & Gammeltoft, S. (2000). A phosphoserine‐regulated docking site in the protein kinase RSK2 that recruits and activates PDK1. The EMBO journal, 19(12), 2924-2934.

Ikeda, M., Ishida, O., Hinoi, T., Kishida, S., & Kikuchi, A. (1998). Identification and characterization of a novel protein interacting with Ral-binding protein 1, a putative effector protein of Ral. Journal of Biological Chemistry, 273(2), 814-821.

Innocenti, M., Frittoli, E., Ponzanelli, I., Falck, J. R., Brachmann, S. M., Di Fiore, P. P., & Scita, G. (2003). Phosphoinositide 3-kinase activates Rac by entering in a complex with Eps8, Abi1, and Sos-1. The Journal of cell biology, 160(1), 17-23.

Jacquemet, G., Green, D. M., Bridgewater, R. E., von Kriegsheim, A., Humphries, M. J., Norman, J. C., & Caswell, P. T. (2013). RCP-driven α5β1 recycling suppresses Rac and promotes RhoA activity via the RacGAP1–IQGAP1 complex. The Journal of cell biology, 202(6), 917-935.

Kandasamy, K., Mohan, S. S., Raju, R., Keerthikumar, S., Kumar, G. S., Venugopal, A. K., ... & Pandey, A. (2010). NetPath: a public resource of curated signal transduction pathways. Genome biology, 11(1), R3.

Kikuchi, A., & Williams, L. T. (1996). Regulation of interaction of ras p21 with RalGDS and Raf-1 by cyclic AMP-dependent protein kinase. Journal of Biological Chemistry, 271(1), 588-594.

King, A. J., Wireman, R. S., Hamilton, M., & Marshall, M. S. (2001). Phosphorylation site specificity of the Pak-mediated regulation of Raf-1 and cooperativity with Src. FEBS letters, 497(1), 6-14.

Li, N. A., Batzer, A., Daly, R., Yajnik, V., Skolnik, E., Chardin, P., ... & Schlessinger, J. (1993). Guanine-nucleotide-releasing factor hSos1 binds to Grb2 and links receptor tyrosine kinases to Ras signalling. Nature, 363(6424), 85-88.

Li, W., Fan, J., & Woodley, D. T. (2001). Nck/Dock: an adapter between cell surface receptors and the actin cytoskeleton. Oncogene, 20(44), 6403-6417.

Matsubara, K., Kishida, S., Matsuura, Y., Kitayama, H., Noda, M., & Kikuchi, A. (1999). Plasma membrane recruitment of RalGDS is critical for Ras-dependent Ral activation. Oncogene, 18(6), 1303-1312.

Montagner, A., Yart, A., Dance, M., Perret, B., Salles, J. P., & Raynal, P. (2005). A novel role for Gab1 and SHP2 in epidermal growth factor-induced Ras activation. Journal of Biological Chemistry, 280(7), 5350-5360.

Nancy, V., Wolthuis, R. M., de Tand, M. F., Janoueix-Lerosey, I., Bos, J. L., & de Gunzburg, J. (1999). Identification and characterization of potential effector molecules of the Ras-related GTPase Rap2. Journal of Biological Chemistry, 274(13), 8737-8745.

Newton, A. (2003). Regulation of the ABC kinases by phosphorylation: protein kinase C as a paradigm. Biochem. J, 370, 361-371.

Okabayashi, Y., Kido, Y., Okutani, T., Sugimoto, Y., Sakaguchi, K., & Kasuga, M. (1994). Tyrosines 1148 and 1173 of activated human epidermal growth factor receptors are binding sites of Shc in intact cells. Journal of Biological Chemistry, 269(28), 18674-18678.

Okada, S., & Pessin, J. E. (1996). Interactions between Src homology (SH) 2/SH3 adapter proteins and the guanylnucleotide exchange factor SOS are differentially regulated by insulin and epidermal growth factor. Journal of Biological Chemistry, 271(41), 25533-25538.

Olayioye, M. A., Neve, R. M., Lane, H. A., & Hynes, N. E. (2000). The ErbB signaling network: receptor heterodimerization in development and cancer. The EMBO journal, 19(13), 3159-3167.

Patel, M., & Karginov, A. V. (2013). Phosphorylation-mediated regulation of GEFs for RhoA. Cell adhesion & migration, 8(1).

Robinson, M. J., & Cobb, M. H. (1997). Mitogen-activated protein kinase pathways. Current opinion in cell biology, 9(2), 180-186.

Rodrigues, G. A., Falasca, M., Zhang, Z., Ong, S. H., & Schlessinger, J. (2000). A novel positive feedback loop mediated by the docking protein Gab1 and phosphatidylinositol 3-kinase in epidermal growth factor receptor signaling. Molecular and cellular biology, 20(4), 1448-1459.

Sarbassov, D. D., Guertin, D. A., Ali, S. M., & Sabatini, D. M. (2005a). Phosphorylation and regulation of Akt/PKB by the rictor-mTOR complex. Science, 307(5712), 1098-1101.

Sarbassov, D. D., Ali, S. M., & Sabatini, D. M. (2005b) Growing roles for the mTOR pathway. Current opinion in cell biology, 17(6), 596-603.

Schlesinger, T. K., Fanger, G. R., Yujiri, T., & Johnson, G. L. (1998). The tao of MEKK. Front Biosci, 3(6), 1181-6.

Scheid, M. P., & Woodgett, J. R. (2003). Unravelling the activation mechanisms of protein kinase B/Akt. FEBS letters, 546(1), 108-112.

Schulze, W. X., Deng, L., & Mann, M. (2005). Phosphotyrosine interactome of the ErbB‐receptor kinase family. Molecular systems biology, 1(1).

Scita, G., Nordstrom, J., Carbone, R., Tenca, P., Giardina, G., Gutkind, S., ... & Di Fiore, P. P. (1999). EPS8 and E3B1 transduce signals from Ras to Rac. Nature, 401(6750), 290-293.

Tamás, P., Solti, Z., Bauer, P., Illés, A., Sipeki, S., Bauer, A., ... & Buday, L. (2003). Mechanism of epidermal growth factor regulation of Vav2, a guanine nucleotide exchange factor for Rac. Journal of Biological Chemistry, 278(7), 5163-5171.

Tolias, K. F., & Cantley, L. C. (1999). Pathways for phosphoinositide synthesis. Chemistry and Physics of Lipids, 98(1), 69-77.

Touré, A., Dorseuil, O., Morin, L., Timmons, P., Jégou, B., Reibel, L., & Gacon, G. (1998). MgcRacGAP, a new human GTPase-activating protein for Rac and Cdc42 similar to Drosophila rotundRacGAP gene product, is expressed in male germ cells. Journal of Biological Chemistry, 273(11), 6019-6023.

Vanhaesebroeck, B., Leevers, S. J., Panayotou, G., & Waterfield, M. D. (1997). Phosphoinositide 3-kinases: a conserved family of signal transducers. Trends in biochemical sciences, 22(7), 267-272.

Wang, Q., Wang, J. Y., Zhang, X. P., Lv, Z. W., Fu, D., Lu, Y. C., ... & Chen, J. X. (2013). RLIP76 is overexpressed in human glioblastomas and is required for proliferation, tumorigenesis and suppression of apoptosis. Carcinogenesis, 34(4), 916-926.
